# Thymidine utilisation pathway is a novel phenotypic switch of *Mycoplasma hominis*

**DOI:** 10.1099/jmm.0.001468

**Published:** 2022-01-17

**Authors:** Gleb Yu. Fisunov, Olga V. Pobeguts, Valentina G. Ladygina, Alexandr I. Zubov, Mariya A. Galyamina, Sergey I. Kovalchuk, Rustam K. Ziganshin, Daria V. Evsyutina, Daria S. Matyushkina, Ivan O. Butenko, Olga N. Bukato, Vladimir A. Veselovsky, Tatiana A. Semashko, Ksenia M. Klimina, Galina A. Levina, Olga I. Barhatova, Irina V. Rakovskaya

**Affiliations:** ^1^​ Department of Molecular Biology and Genetics, Federal Research and Clinical Centre of Physical–Chemical Medicine, Moscow, Russia; ^2^​ Shemyakin–Ovchinnikov Institute of Bioorganic Chemistry, Russian Academy of Sciences, Moscow, Russia; ^3^​ Department of Biotechnology, Vavilov Institute of General Genetics Russian Academy of Sciences, Moscow, Russia; ^4^​ Gamaleya National Research Center of Epidemiology and Microbiology, Moscow, Russia

**Keywords:** *Mycoplasma hominis*, micro-colonies, proteomic analysis, nucleoside catabolism, thymidine phosphorylase, resistant to antibiotics

## Abstract

**Introduction:**

*
Mycoplasma hominis* is a bacterium belonging to the class *Mollicutes *. It causes acute and chronic infections of the urogenital tract. The main features of this bacterium are an absence of cell wall and a reduced genome size (517–622 protein-encoding genes). Previously, we have isolated morphologically unknown *M. hominis* colonies called micro-colonies (MCs) from the serum of patients with inflammatory urogenital tract infection.

**Hypothesis:**

MCs are functionally different from the typical colonies (TCs) in terms of metabolism and cell division.

**Aim:**

To determine the physiological differences between MCs and TCs of *M. hominis* and elucidate the pathways of formation and growth of MCs by a comparative proteomic analysis of these two morphological forms.

**Methodology:**

LC–MS proteomic analysis of TCs and MCs using an Ultimate 3000 RSLC nanoHPLC system connected to a QExactive Plus mass spectrometer.

**Results:**

The study of the proteomic profiles of *M. hominis* colonies allowed us to reconstruct their energy metabolism pathways. In addition to the already known pentose phosphate and arginine deamination pathways, *M. hominis* can utilise ribose phosphate and deoxyribose phosphate formed by nucleoside catabolism as energy sources. Comparative proteomic HPLC–MS analysis revealed that the proteomic profiles of TCs and MCs were different. We assume that MC cells preferably utilised deoxyribonucleosides, particularly thymidine, as an energy source rather than arginine or ribonucleosides. Utilisation of deoxyribonucleosides is less efficient as compared with that of ribonucleosides and arginine in terms of energy production. Thymidine phosphorylase DeoA is one of the key enzymes of deoxyribonucleosides utilisation. We obtained a DeoA overexpressing mutant that exhibited a phenotype similar to that of MCs, which confirmed our hypothesis.

**Conclusion:**

In addition to the two known pathways for energy production (arginine deamination and the pentose phosphate pathway) *M. hominis* can use deoxyribonucleosides and ribonucleosides. MC cells demonstrate a reorganisation of energy metabolism: unlike TC cells, they preferably utilise deoxyribonucleosides, particularly thymidine, as an energy source rather than arginine or ribonucleosides. Thus MC cells enter a state of energy starvation, which helps them to survive under stress, and in particular, to be resistant to antibiotics.

## Introduction

*Mcoplasma hominis* is an opportunistic pathogen that causes acute and chronic infections of the urogenital tract in humans [1]. Intrauterine *M. hominis* infections can cause meningitis, pneumonia or abscesses in neonates [1]. *M. hominis* also causes acute and chronic pyelonephritis [2]. The mycoplasma adheres to blood cells, spreads to organs and tissues and causes generalised mycoplasma infections [3, 4]. Another feature of this bacterium is its long-term persistence in the tissues of infected organisms [5]. This bacterium, like all other mycoplasmas, is characterised by an absence of a cell wall, a reduced genome size (570 protein-encoding genes for strain H-34), and reduced metabolic pathways. Previously, we have isolated bacterial colonies of a previously unknown morphological type by the culture method from the blood serum of patients with urogenital tract infections. The bacteria were identified as representatives of the genus *Mycoplasma* [6, 7]. In most cases, *M. hominis* was detected; however, *Mycoplasma pneumoniae* and *Mycoplasma fermentans* were detected occasionally. We termed them ‘micro-colonies’ (MCs).The isolated cultures were identified using species-specific tests. In the case of *M. hominis *, MCs tests are positive by PCR with primers specific for *M. hominis *, direct fluorescence and epifluorescence tests, and Western blotting with aMh-FcG2a nanobodies, derived from a camel, which are specific for the MH3620 transporter protein [7]. Similar MCs were obtained from an *in vitro* laboratory system under unfavourable conditions. Treatment with antibodies, various types of non-thermal plasma, cultivation in a poor medium and long-term incubation (at 37 °C for over 2 weeks) were considered as unfavourable factors [7, 8]. We also showed that a pure culture of MCs can be obtained without treatment. Using a light microscope, the growth of a clonal culture *M. hominis* on solid agar was monitored for 12 days. The appearance of typical colonies (TCs) was observed in 24–48 h, on the fifth day, part of the TCs underwent lysis and MCs appeared, the number of which increased to the 12th day of observation. On the 12th day of growth, we observed only MC. It is possible that the initial population of mycoplasmas is heterogeneous and consists of cells devoted to the formation of both TCs and MCs. However, it is also possible that there is a transition from one phenotype to another under the affect of unfavourable factors. The reverse transition of cells from MCs to TCs was not observed, which indicates the presence of stable switching. The latter hypothesis raises questions about the underlying mechanisms that determine the MC phenotype.

Previously, we demonstrated that MCs differ from TCs in the following ways:

1. Morphology: TCs have a rounded shape, reminiscent of a fried egg while MCs consist of a bundle of helical rays protruding from the centre of the colony.
2. Size difference: The diameter of TСs was 100–300 µm and that of MCs was 7–9 µm.
3. Growth rates: TCs grow on an agar surface for 48–72 h while primary MCs grow on agar for 7–12 days.
4. Cell division: MCs do not grow on a liquid medium.
5. Carbon source: Unlike TCs, MCs can grow and proliferate on a medium without arginine or glucose.
6. Resistance: MCs are extremely resistant to unfavourable factors such as antibiotics and prolonged starvation [7, 8]. The results of antibiotic susceptibility testing of TCs and MCs using the disc diffusion method indicated that MCs were resistant to a range of antibiotics (gentamicin, norfloxacin, doxycycline, clarithromycin, roxithromycin, clindamycin, azithromycin, erythromycin and lincomycin), while TCs were sensitive to all these antibiotics except erythromycin.

It is likely that small colony sizes and very slow growth rates have hidden MCs from being observed previously. We propose that a novel form of *M. hominis* can persist in humans. This form is resistant to unfavourable factors and is invisible to the host immune system. This form is functionally different from the well-characterised form of *M. hominis* in terms of metabolism and cell division. In order to reveal the mechanistic basis of the formation and growth of *M. hominis* MCs, we performed comparative proteomic analysis of TCs and MCs, which were obtained after treatment of *M. hominis* strain H-34 with hyperimmune serum.

## Methods

### Bacterial strain and growth conditions

*M. hominis* strain H-34 was kindly provided by Dr K.H. Lemcke (Listar Instute of Preventive Medicine, London, UK). *M. hominis* strain H-34 was cultivated on BBL Mycoplasma agar (PPLO Agar base, BD) and in Difco PPLO Broth (BD) supplemented with 15 % horse serum (Gibco, Thermo Fisher Scientific), 2.5 % yeast extract, 1 % l-arginine or 20 mM thymidine as the sole carbon source. Penicillin was added up to 100 µg ml^−1^ to avoid contamination. Colonies that appeared on BBL Mycoplasma agar plates were observed with the light microscope with 10×, 25× and 40× objectives (LETZLAR). TCs were counted 96 h post-plating. MCs were counted when they became clearly visible, usually on the seventh to ninth day. To re-plate MCs we applied a ‘block method’. Briefly, a cube of agar with a side of 1 cm was cut off from the plate with MCs. To evenly distribute bacteria, the cube was applied upside down to a fresh plate with a light circular motion.

The increase in the gDNA level was used as a measure to obtain growth curves of a liquid culture of *M. hominis* H-34. At regular intervals, 1 ml of mycoplasma culture was taken. DNA was isolated according to a method described by us previously [9].

### Mycoplasma treatment with hyperimmune serum

The hyperimmune serum was developed by rabbit immunisation with mycoplasma membrane fractions that were obtained as described previously [10]. Immunisation of 2.5–3 kg rabbits was performed according to the standard scheme [11]. The treatment of mycoplasma with the hyperimmune serum was performed as described previously [7]. Pure MC cultures were generated *in vitro* by treatment of mycoplasma cultures with hyperimmune serum. Full rabbit serum was added to the bacterial suspension (10^7^ c.f.u.) in the ratio 1 : 1. The mixture was incubated at 37 °C for 24 h. Then decimal dilutions were plated on BBL Mycoplasma agar. MCs grew 12 days after coating.

### Antibiotic test

The sensitivity of TCs, MCs and a DeoA:TnRM5 mutant to antibiotics was analysed using the agar dilution method [12]. Gentamicin, clarithromycin and ofloxacin were used as antibiotics. Antibiotic agar plates were prepared as described in the Antimicrobial Susceptibility Testing Methods Manual for Human Mycoplasmas [12]. TC and MC colonies were washed off the agar (five plates for TCs and 15 plates for MCs) using Difco PPLO Broth, centrifuged for 10 min at 10 000 ***g*** and 4 °C. The pellets were resuspended in 1.5 ml of the same medium and used for plating on BBL Mycoplasma agar plates. The DeoA:TnRM5 mutant was grown to the exponential phase of growth in Difco PPLO Broth, supplemented with 15 % horse serum, 2.5 % yeast extract and 20 mM thymidine as the sole carbon source, diluted ten times and seeded on agar plates. At least three agar plates were used for each antibiotic concentration. Colony growth was observed using a microscope (LETZLAR). The number of colonies was counted as the average, counted in five fields of view in three agar plates for each condition. In the case of TCs, colonies were counted after 3 days and 7 days of growth, and in the case of MCs and DeoA:TnRM after 7 days of growth.

### Free-gel digestion of protein samples

Colonies were washed off the agar with 50 mM Tris-HCl buffer (pH 7.4) containing 150 mM NaCl and 5 mM MgCl_2_ (buffer A). To collect enough micro-colonies for the study, we had to use a large number of agar plates for every repeat (30 agar plates for MCs and 5–10 agar plates for TCs). This was due to the very small colony size, their deeper growth into agar compared with TCs, and high losses in the process of protein purification and extraction. Each suspension was centrifuged at 1000 ***g*** at 4 °C for 2 min to remove residual agar. The supernatant was centrifuged at 10 000 ***g*** at 4 °C for 20 min. Since a significant amount of nutrient medium proteins got into the sample, the washings of the colonies were passed twice through an isotonic sucrose solution to cleanse the cells from contamination. For this the resulting pellet was resuspended in buffer A, and was centrifuged twice using a 0.25 M sucrose cushion at 10 000 ***g*** at 4 °C for 30 min to remove proteins from the culture medium. The degree of purification from contamination was monitored using the HPLC–MS method by identifying eukaryotic proteins in the samples. Further, 10 µl of 10 % sodium deoxycholate (DCNa) and 0.5 µl nuclease mix (GE Healthcare) were added to the cell pellet. After incubation at 4 °C for 1 h, the samples were resuspended in 100 µl of 100 mM Tris–HCl buffer (pH 8.5) containing 0.1 % DCNa, 8 M urea, and 2.5 mM ethylenediaminetetraacetic acid. After incubation for 20 min, the samples were centrifuged at 14 000 ***g*** at 4 °C for 10 min to remove intact cells and debris. The supernatant was collected, and the protein concentration was measured using a Bradford Assay Kit (BioRad). Disulphide bonds were reduced in the supernatant containing 100 µg of total protein by the addition of tris(2-carboxyethyl) phosphine hydrochloride (TCEP) (Sigma) to a final concentration of 5 mM, and the reaction was incubated at 37 °C for 60 min. To alkylate free cysteines, chloroacetamide (BioRad) was added to a final concentration of 30 mM, and the solution was placed at room temperature (RT) in the dark for 30 min. The TCEP addition was repeated. The sample was diluted sixfold with 50 mM Tris-HCl (pH 8.5) and 0.01 % DCNa Trypsin (Trypsin Gold, Mass Spectrometry Grade, Promega) to achieve a final trypsin:protein ratio of 1 : 50 (w/w), and it was subsequently incubated at 37 °C overnight. To prevent trypsinolysis and to degrade the acid-labile DCNa, trifluoroacetic acid (TFA) was added to a final concentration of 0.5 % (v/v) (the pH should be less than 2.0), and the samples were incubated at 37 °C for 45 min. The samples were centrifuged at 14 000 ***g*** for 10 min to remove DCNa. The peptide extract was desalted using a Discovery DSC-18 tube (Supelco) according to manufacturer’s protocol. Peptides were eluted with 1 ml of 75 % acetonitrile (ACN) solution containing 0.1 % TFA, dried in a SpeedVac (Labconco), and resuspended in a 3 % ACN solution containing 0.1 % TFA to a final concentration of 5 µg µl^−1^. Samples of TCs and MCs were subjected to the same degree of washing and digestion.

### LC–MS analysis

Before analysis, the samples were aligned for the amount of protein. LC–MS analysis was conducted using an Ultimate 3000 RSLC nanoHPLC system connected to a QExactive Plus mass spectrometer (Thermo Fisher Scientific). Samples were loaded onto a home-made trap column (20×0.1 mm), packed with Inertsil ODS-3 3 µm sorbent (GLSciences) in the loading buffer (2 % ACN, 98 % H_2_O, 0.1 % TFA) at a flow rate of 10 ml min^−1^, separated at RT in a home-made fused-silica column (500×0.1 mm) packed with ReproSil-Pur C18-AQ 1.9 (Dr Maisch), and discharged into the emitter through a P-2000 laser puller (Sutter) [2]. Samples were eluted using a linear gradient of 80 % ACN, 19.9 % H_2_O, and 0.1 % formic acid (solvent B) and 99.9 % H_2_O and 0.1 % formic acid (solvent A) by increasing the concentration of solvent B from 4–36 % in 1 h at a flow rate of 0.44 µl min^−1^ at RT.

The mass spectrometer was operated in data-dependent acquisition mode, and the MS1 parameters were as follows: 70K resolution, scan range: 350–2000, maximum injection time: 50 ms, and AGCtarget: 3×10^6^. Ions were isolated in a 1.4 m/z window with a 0.2 m/z offset targeting the ten highest intensity peaks between the charges of +2 to+6, having a minimum AGC target of 8×10^3^. Preferred peptide match and isotope exclusion was conducted along with dynamic exclusion, which was set to 40 s. MS2 fragmentation was carried out in higher energy collisional dissociation mode with a 17.5K resolution at 27 % NCE. Ions were collected for a maximum of 45 ms with a target AGC of 1×10^5^.

### Protein identification and quantification

Label-free quantification and identification of proteins were performed with the MaxQuant 1.6.6.0 software (https://www.maxquant.org/)using default settings. Data were searched against the *M. hominis* ATCC 23114 NCBI database. The *M. hominis* ATCC 23114 complete genome assembly was used as a reference, but it can limit identification results due to possible genomic differences between strains ATCC 23114 and H-34.

### Construction of DeoA- (DeoA:TnRM5) and EGFP- (EGFP:TnRM5) overexpressing vectors

To construct *M. hominis* overexpressing *deoA* and *egfp* genes, we used a transposon vector (pMSC-M5) with a strong constitutive promoter and optimal ribosome-binding site. The transposon-based vector is shown schematically in Fig. S1 (available in the online version of this article). The *deoA* gene sequence was amplified from *M. hominis* genomic DNA using the following primers: deoA_mho_pRM5_F TATACTCGAGATGAGAATTATTGATATTATTAACAAAAAAGTAG and deoA_mho_pRM5_R TAATCCATGGTTACATTAATTTAGCAAAAACTGTTTG. Polymerase chain reaction was performed using Phusion High-Fidelity DNA Polymerase (Thermo Fisher Scientific). The PCR product was purified using chloroform extraction and isopropanol precipitation, cut with *XhoI* and *NcoI* restriction enzymes (Thermo Fisher Scientific), and ligated into the pMSC-M5 vector between the *XhoI* and *NcoI* restriction sites using T4 DNA ligase (Thermo Fisher Scientific). The ligated product was transformed into TOP10 Chemically Competent *Escherichia coli* (Thermo Fisher Scientific), and plated onto Luria–Bertani (LB)/ampicillin medium. Colonies were selected, screened for insertion by PCR, enriched in LB/ampicillin medium and partially sequenced. The sequences of inserted fragments were obtained by the Sanger dideoxy sequencing method using Bigdye Terminator v.3.1 Cycle Sequencing Kit and ABI Prism Genetic Analyzer 3730XL following the manufacturer’s instructions (Applied Biosystem). The correct plasmid was transformed into *M. hominis *. The transformation of *M. hominis* was performed using electroporation as described previously for *M. gallisepticum* [13]. The pMSC-M5 plasmid with an *egfp* gene had been obtained previously [14]. After electroporation, bacterial cells were transferred into fresh arginine-supplemented antibiotic-free liquid medium and cultured for 4 h at 37 °C. Then, the cells were plated on semi-liquid arginine-supplemented medium with tetracycline (2 µg ml^−1^) for identification and recovery of successful transformants. Five colonies for each plasmid were picked and enriched. The transposon insertions were confirmed and mapped by PCR and Sanger sequencing from the chromosome as described previously [15].

### RNA purification and cDNA synthesis

RNA was isolated as previously described [9]. Aliquots (100 µl of *M. hominis* culture were directly lysed in TRIzol LS reagent (Life Technologies) at a 1 : 3 ratio of culture medium: TRIzol LS (v/v). The nucleic acids were extracted with chloroform and precipitated by the addition of an equal volume of isopropanol followed by centrifugation. The pellets were washed with 80 % ethanol and finally resuspended in 20 µl mQ (Panreac). The amount of RNA was determined using a Qubit 2.0 fluorometer (Thermo Fisher Scientific). The resulting RNA was treated with DNAse I (Thermo Scientific), and cDNA was synthesised from random hexamer primers by using Maxima H Minus Reverse Transcriptase (Thermo Scientific) according to the manufacturer’s protocol.

### qRT-PCR

Quantitative real-time PCR was performed using dNTP, PCR buffer, Taq-polymerase (Lytech), SYBR Green I (Invitrogen), and CFX96TM Real-Time PCR Detection System (Bio-Rad). The primers used are listed in Table S1. Each 20 -µl reaction contained 0.2 µl of template cDNA. The thermal cycling conditions were as follows: initial denaturation at 95 °C for 1 min; then 40-cycle amplification (94 °C for 15 s, 58 °C for 20 s, and 68 °C for 1 min) with a single fluorescence per reading. The melting curve was obtained by gradually heating the PCR mixture from 65–94 °C at a rate of 0.5 °C every 5 s, with continuous fluorescence scanning. Relative expression values were calculated as 2^−(CtTarget − CtReference)^, where Ct is the fractional threshold cycle for the target gene and the reference is the *tuf* (elongation factor Tu) mRNA. qRT-PCR experiments were performed on three biological replicates per transformant. The significance of the difference between the two mean values was assessed using the Wilcoxon rank test implemented in the R software package. *P*<0.05 (*); ns, no significant difference.

### Genome sequencing

Genomic DNA of *Mycoplasma hominis* deoA:TnRM5 and WT-H34 strains was extracted by the PureLink Genomic DNA Mini Kit (Thermo Fisher Scientific). Extracted DNA was disrupted into 200–300 bp fragments using the Covaris S220 System (Covaris). The library was constructed using the NEBNext Ultra II DNA Library Prep Kit for Illumina (NEB). Whole-genome sequencing (paired-end) was carried out on the Illumina MiSeq platform (Illumina). After the removal of adapters and low-quality reads, sequencing reads were assembled using the SPAdes genome assembler [16]. The obtained genome sequences were annotated using PROKKA [17]. The draft genomes will be available in GenBank as soon as they are processed by the service, BioSample accession numbers: SAMN20983040 and SAMN20983041. Raw sequencing data were deposited in the NCBI Sequence Read Archive (SRA), accession numbers: SRR15616379 and SRR15616380 (https://www.ncbi.nlm.nih.gov/Traces/study/?acc=PRJNA757936&o=acc_s%3Aa).

### Differential 2D gel-electrophoresis (2D-DIGE), tryptic digestion and protein identification

2D-DIGE, tryptic digestion of the proteins and protein identification by MALDI-ToF mass-spectrometry were performed as described above [15]. The gels were scanned on a Typhoon Trio (Amersham) scanner at 532 nm (Cy3) and 633 nm (Cy5) PMT 500 V laser intensity. Quantitative analysis was performed using PDQuest 8.0 software (Bio-Rad). For spot excision the gels were stained with silver as described by Shevchenko *et al*. [18]. The spots of interest were excised and washed in 30 mM of sodium thiosulphate and 100 mM of potassium ferricyanide at a 1 : 1 ratio. Then the gel pieces were washed in mQ water until the yellow colour disappeared. Then gel pieces were dried in 100 % acetonitrile. A 3–4 µl volume of trypsin solution (40 mM ammonium bicarbonate, 10 % acetonitrile, 40 nM trypsin) was added to each sample and the samples were incubated for 30 min on ice and subsequently for 16–17 h at 37 °C. Peptides extraction was performed by addition of 0.5 % v/v TFA in mQ water. The samples were incubated in an ultrasonic bath for 10 min and then incubated for 1 h at room temperature. Mass-spectrometric analysis was performed on Ultraflex II MALDI-ToF-ToF (Bruker Daltonics) as described previously [19]. Protein identification was carried out by a peptide fingerprint search with the use of Mascot software (Matrix Science) through a NCBI protein *M. hominis* ATCC 23114 NCBI database. The identification cutoff was 44 (*P*<0.05).

### Statistical analysis

Descriptive statistics of mean values, standard deviation (SD) and standard error (SE), confidence intervals (CI), Welch ANOVA, Shapiro-Wilk and F-tests were performed using R-3.6.3.

## Results

### Micro-colonies differ from typical colonies in physiology and proteome

In order to determine the basis of physiological differences between TCs and MCs, we carried out comparative proteomic analyses. Since MC cells do not grow in a liquid medium, colonies of both morphological types were grown on solid agar. Colonies were washed off the agar as described in the methods. In order to collect enough micro-colonies for research, we had to use a large number of agar plates (30 agar plates for MCs and 5–10 agar plates for TCs). This was due to the very small colony size, their deeper growth into agar compared with TCs and high losses in the process of protein purification and extraction. Since a significant amount of nutrient medium proteins entered the sample, the washings of the colonies were passed twice through an isotonic sucrose solution to cleanse the cells from contamination. The degree of purification from contamination was monitored using the HPLC-MS method by identifying eukaryotic proteins in the samples. Before analysis, the samples were aligned for the amount of protein. Three biological replicates were analysed using the Max Quant 1.6.6.0.software (OP1, OP2 and OP3). The criterion for protein identification was detection of at least two peptides. The number of identified proteins were 448 (OP1), 220 (OP2), 293 (OP3) and 449 (OP1), 112 (OP2), 198 (OP3) for the three biological replicates of TCs and MCs, respectively. Among them, 288 (OP1), 117 (OP2) and 76 (OP3) differentially expressed proteins were present in three replicates. Label-free quantification was used to determine the relative amounts of proteins present in the replicates (Table S2). Of the total number of identified proteins in TCs and MCs, common to the three replicates, the abundance of 165 (OP1), 41 (OP2), 46 (OP3) proteins decreased (with a log2FC cut-off of −0.5) and 4, 25 and 14 proteins increased (with a log2FC cut-off of 0.5). We noted that the trend of changes is similar in all three biological repeats.

**Fig. 1.**
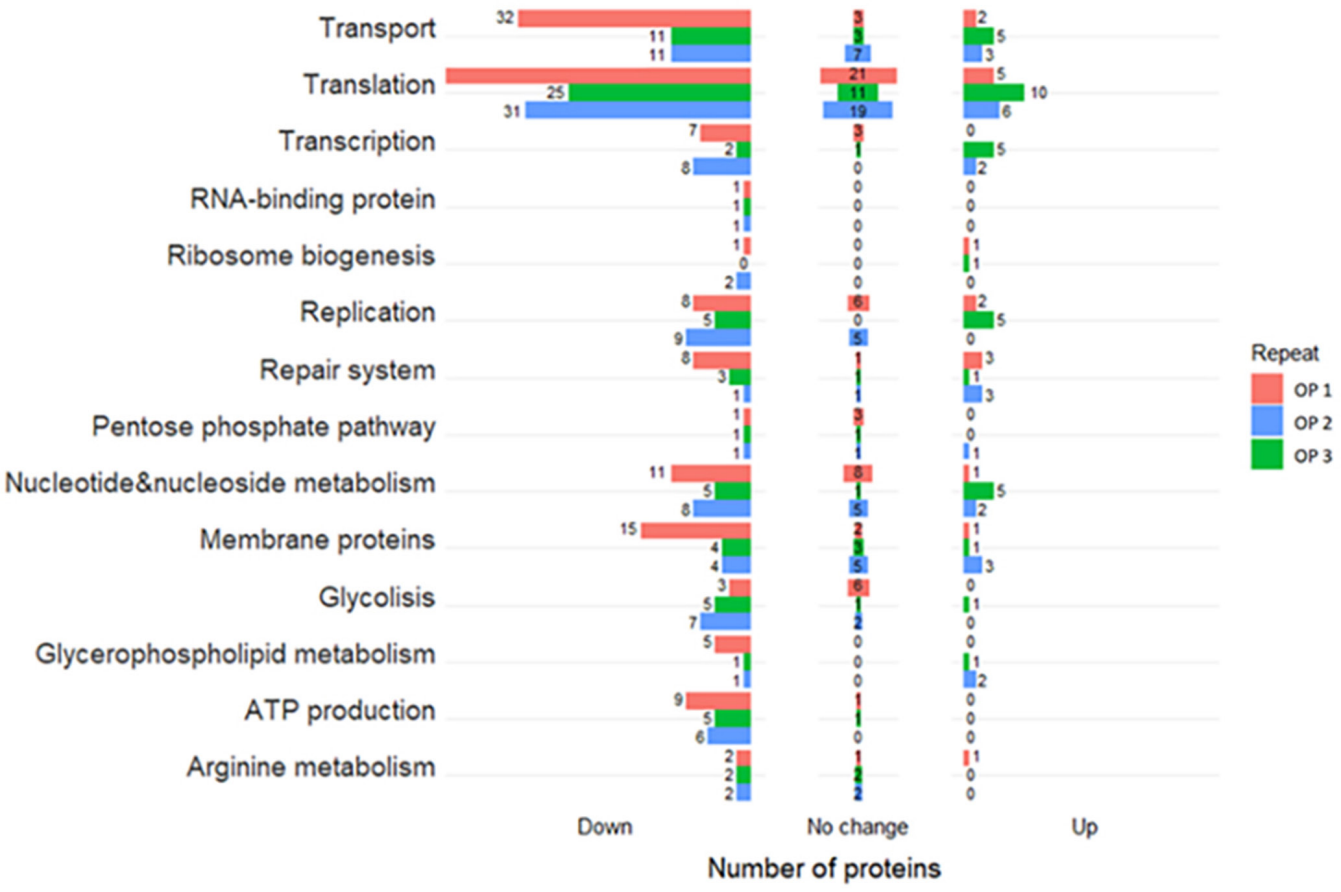
Functional characteristic of differentially changing proteins in MCs relative to TCs of *M. hominis* in three biological replicates (OP1, OP2 and OP3). The first replicate (OP1) highlighted in red, the second replicate (OP2) highlighted in green and the third replicate (OP3) highlighted in blue. The numbers indicate the amounts of upregulated (right), downregulated (left) and unchanged proteins (middle).

The downregulated proteins perform a broad range of functions including transcription, translation, ATP production and membrane transportation (Table S2). The abundance of sigma factor decreased in two biological replicates. Additionally, there was a decrease in the abundance of enzymes involved in glycerophospholipid metabolism, nucleotide metabolism (phosphopentomutase DeoB, purine-nucleoside phosphorylase DeoD) and energy metabolism pathways, such as glycolysis (Fba, Pgi) and arginine dihydrolase pathways [arginine deiminase (ArcA) and *N*-dimethylarginine dimethylaminohydrolase (DDAH)] (Tables 1 and 2). Thymidine phosphorylase (DeoA), an enzyme involved in nucleoside catabolism, was upregulated in all three replicates. Micro-colonies were obtained *in vitro* by treating *M. hominis* strain H-34 with hyperimmune serum [7]. The treatment of mycoplasma with antibodies leads to a change in the repertoire of variable surface lipoproteins [20, 21]. Lmp-family proteins, P120, P75, OppA, OppF, MHO_0730, and MHO_3200, which are involved in adhesion and host–pathogen interactions, were downregulated in MCs whereas the Lmp3 protein was upregulated in one replicate (not identified in the other two). This may indicate antigenic variation, a common immune escape strategy in mycoplasmas [22, 23]. An overview of the functional changes is shown in Fig. 1. Although there are multiple changes in protein expression, it is important to identify those that are directly involved in the transition of TCs to MCs.

**Table 1.** Changes of the enzymes of energy metabolism in MCs compared with TCs for three biological replicates, calculated using LC–MS analysis and label-free identification with MaxQuant 1.6.6.0 (MCs/TCs LC–MS colonies), as well as in liquid culture of *M. hominis* H-34 grown arginine (WT H-34 arginine) compared with a DeoA:TnRM5 mutant with overexpression of the *deoA* gene grown on media with thymidine, calculated using 2D differential electrophoresis and PDQuest 8.0 software (2D suspension culture WT H-34 arginine/DeoA:TnRM5 thymidine)

Protein ID	Gene	Protein	MCs/ TCs LC–MS colonies biological replicate 1	MCs/ TCs LC–MS colonies biological replicate 2	MCs/ TCs LC–MS colonies biological replicate 3	2D Liquid culture of WT H-34 arginine/ DeoA:TnRM5 thymidine
**Arginine-dehydrolase pathway**						
D1J7K6	*arcA*	Arginine deiminase	0.5	0.55	0.34	0.33±0.04
D1J7K1	*arcB*	Ornithine carbamoyl transferase	0.75	120	0.95	–
D1J7K0	*arcC*	Carbamate kinase	1.56	1.20	0.74	0.44±0.06
D1J841		*N*-dimethylarginine dimethylaminohydrolase	0.5	only TCs	0.46	–
**Glycolysis**						
D1J7M6	*eno*	Enolase	0.78	only TCs	1.14	0.51±0.07
D1J8K6	*pyk*	Pyruvate kinase	0.72	0.60	0.43	–
D1J7I6	*pgk*	Phosphoglycerate kinase	1.22	only TCs	0.43	0.42±0.05
D1J8E6	*ldh*	l-lactate dehydrogenase	1.21	0.77	0.70	0.15±0.02
D1J8V2	*gap*	Glyceraldehyde 3-phosphate dehydrogenase	0.64	4.16	0.71	0.11±0.01
Q6Y8Q8	*gpmI*	2,3-bisphosphoglycerate-independent phosphoglycerate mutase	0.95	–	only TCs	–
	*fba*	Fructose-bisphosphate aldolase	0.5	–	only TCs	–
D1J8E7	*pgi*	Glucose-6-phosphate isomerase	0.75	only TCs	0.54	–
**Pentosephosphate pathway**						
D1J8E3	*prs*	Ribose-phosphate pyrophosphokinase	0.63	1.32	0.75	–
D1J8H6	*rpiB*	Ribose-5-phosphate isomerase	1.26	–	–	–
D1J7W4	*tktA*	Transketolase	0.81	–	1,73	–
D1J889	MHO_3010	Phosphoketolase	0.76	0.48	0.43	–

**Table 2.** Changes of nucleoside metabolism enzymes in MCs compared with TCs for three biological replicates, calculated using LC–MS analysis and label-free identification with MaxQuant 1.6.6.0 (MCs/TCs LC–MS colonies), as well as in liquid culture of *M. hominis* H-34 grown in media with arginine (WT H-34 arginine) compared with DeoA:TnRM5 mutant with overexpression of *deoA* gene grown in media with thymidine, calculated using 2D differential electrophoresis and PDQuest 8.0 software (2D suspension culture WT H-34 arginine/DeoA:TnRM5 thymidine)

Protein ID	Gene	Protein	MCs/ TCs LC–MS Colonies biological replicate 1	MCs/ TCs LC–MS colonies biological replicate 2	MCs/ TCs LC–MS colonies biological replicate 3	2D Liquid culture WT H-34 arginine/ DeoA::TnRM5 thymidine
D1J8C2	*deoA*	Thymidine phosphorylase	1.70	1.55	10.55	2.75±0.30
D1J8C1	*deoC*	Deoxyribose-phosphate aldolase	1	only MCs	0.92	4.09±0.53
D1J8K5	*deoB*	Phosphopentomutase	0.57	0.28	0.53	0.45±0.06
D1J8C3	*deoD*	Purine-nucleoside phosphorylase	0.35	1.12	0.70	–
D1J7L3	MHO_0760	putative glycerol-3-phosphate specific transporter	0.40	only MCs	–	–
D1J7W3	MHO_1760	alcohol dehydrogenase	0.32	0.53	–	–
D1J8U2	MHO_5040	putative arginine transporter	0.44	2.56	1.35	–

### Metabolic reconstructions of *M. hominis* TCs and MCs and detection of a key stage in MC formation

**Fig. 2.**
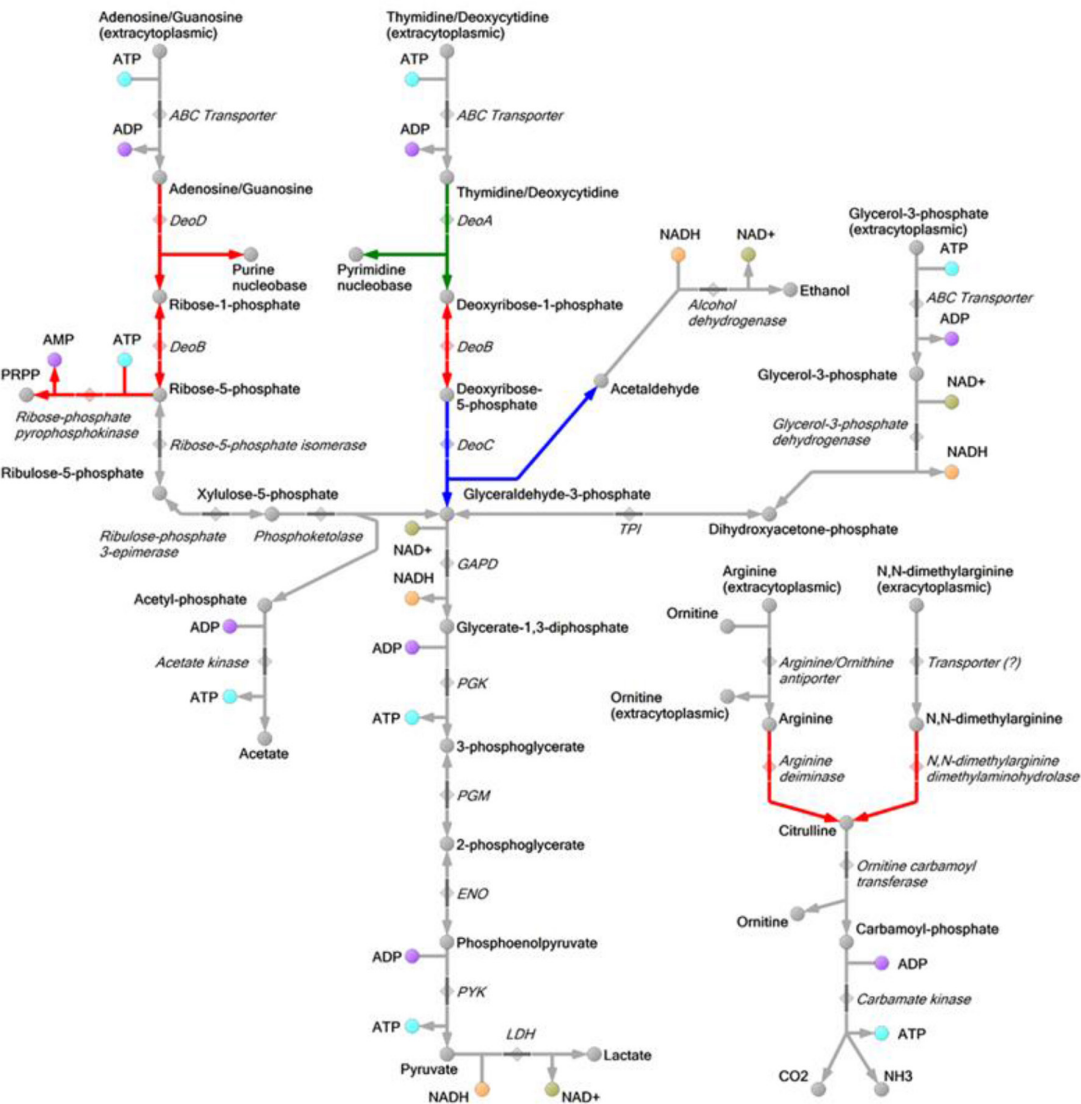
Reconstructed map of energy metabolism of *M. hominis *. The upregulated reactions are highlighted in green, downregulated in red and unchanged in blue.

**Fig. 3.**
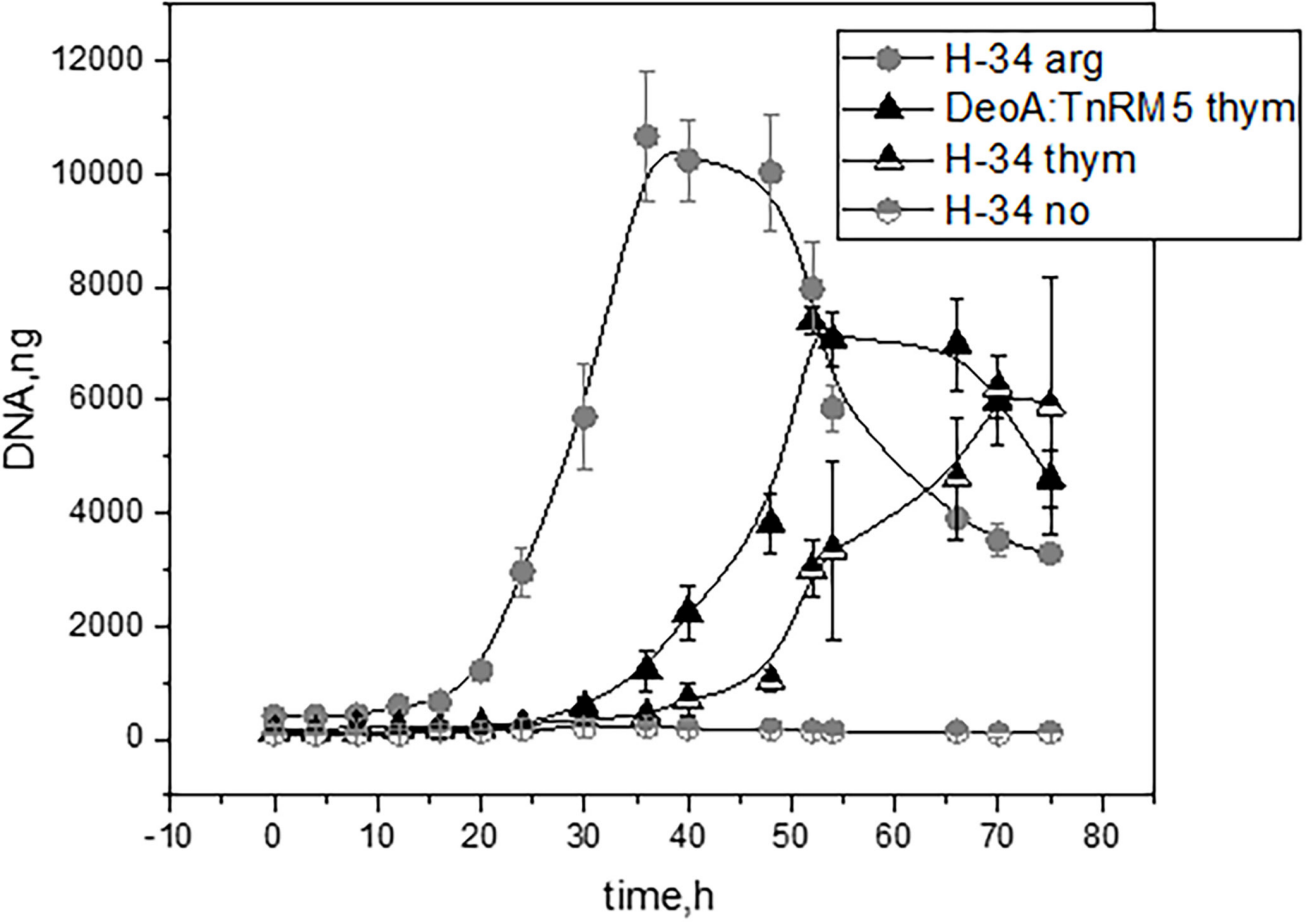
Growth curves of the wild type (WTH-34) grown on media with arginine (H-34 arg), with thymidine (H-34 thym) and without carbon source (H-34 no), and mutant (DeoA:TnRM5) of *M. hominis* grown on media with thymidine (DeoA:TnRM5 thym).

The significantly reduced genomes of mycoplasmas decrease their synthetic capacities. Generally, they retain their energy metabolism and at the same time consume a majority of nutrients from the host. The energy metabolism of *M. hominis* is poorly understood. There are two major pathways for energy production: arginine deamination and the pentose phosphate pathway (PPP) [24, 25]. The glycolytic pathway of *M. hominis* lacks the key enzyme 6-phosphofructokinase, and thus it is incomplete. The phosphotransferase system (PTS) is also incomplete, and it comprises only HPr- and EIIB-encoding genes annotated in the genome. However, downstream enzymes and some components of PPP are present. In *M. hominis *, PPP is also critically incomplete, containing only isomerases and transketolases [24, 25]. The lack of sugar kinases or functional PTS does not allow *M. hominis* to utilise free carbohydrates. Enzymes of the glycolytic pathway and PPP along with phosphoketolase and deoxyribose-phosphate aldolase (DeoC) enable the utilisation of ribose-phosphate and deoxyribose-phosphate produced by nucleoside catabolism. The respective metabolic reconstructions are shown in Fig. 2. The utilisation of ribose-phosphate produces three molecules of ATP, whereas the utilisation of deoxyribose-phosphate produces two molecules of ATP per molecule of nutrient. However, the transport of nucleosides mainly occurs through ABC transporters, and thus, it consumes ATP. The ATP/substrate stoichiometry for the transport of nucleosides by ABC transporters is crucial, and it is unclear for *M. hominis *. Generally, it can vary from 1 : 1 [26] to 2 : 1 [27], and can also be a fractional value. For example, for the maltose transporter of *E. coli *, the ATP/substrate stoichiometry is 1.4 : 1 [28]. For deoxyribonucleosides, the ratio should be less than two; otherwise, the respective metabolic pathway does not produce ATP. Genomic analysis of *M. hominis* revealed the absence of ribonucleoside reductase. Hence, there is no possibility of an interconversion between ribonucleosides and deoxyribonucleosides. To analyse the ability of *M. hominis* to use deoxyribonucleosides as an energy source, we cultured *M. hominis* on thymidine instead of arginine. We observed that *M. hominis* grew at a significantly lower rate on thymidine than on arginine (Fig. 3). It was also able to grow on guanosine (date not shown). We found a putative glycerol-3-phosphate specific transporter in *M. hominis* (MHO_0760) [25]. Glycerol-3-phosphate can be used as an energy source, but its utilisation depends on the efficiency of the transport system, similar to that of deoxyribonucleosides. If the utilisation of glycerol-3-phosphate produces two molecules of ATP, and the same number of ATP are spent in its transport, the utilisation of glycerol-3-phosphate is not more efficient than that of deoxyribonucleosides in terms of energy output. Another problem with the utilisation of glycerol-3-phosphate is the production of NADH, which has to be spent. Our metabolic reconstruction predicts that the spare NADH can be used to reduce acetaldehyde (produced by DeoC) to ethanol by a putative alcohol dehydrogenase (MHO_1760). Thus, the utilisation of deoxyribonucleosides may boost the utilisation of glycerol-3-phosphate as an energy source.

Arginine deamination results in the production of one molecule of ornithine and one molecule of ATP per molecule of arginine. The transport of arginine probably occurs through an arginine/ornithine antiporter, which does not consume ATP [25]. A putative arginine transporter (MHO_5040), containing 12 transmembrane domains, was identified as a homologue of the amino acid permease MYPE6110. MYPE6110 is encoded by a gene located downstream of the *arcABDC* gene cluster, which encodes the arginine deiminase pathway in *Mcoplasma penetrans* [29], another arginine-utilizing mycoplasma. Additionally, there are genes encoding a putative Opp ABC transport system (MHO_1510–1550) in *M. hominis *. Therefore, arginine-containing oligopeptides should be considered as possible alternative sources of arginine.

Thus, the utilisation of arginine results in the net production of one molecule of ATP per molecule of arginine. For ribonucleosides, the net ATP produced ranges from one to two while for deoxyribonucleosides, the net ATP produced is no more than 1 but above zero. Hence the utilisation of ribonucleosides is the most efficient process in terms of energy production, followed by arginine utilisation, while deoxyribonucleosides utilisation is the least efficient.

The MC state of *M. hominis* is characterised by the downregulation of two out of four enzymes involved in the pathway of arginine (Table 1). Thymidine phosphorylase (DeoA) is among the few proteins upregulated in MC (Table 2), and it catalyses the first step in the utilisation of pyrimidine deoxyribonucleosides to produce energy. Simultaneously, purine nucleoside phosphorylase (DeoD), which is the first enzyme in the utilisation of ribonucleoside, was downregulated (Table 2). Thus, we suggested that there is a reorganisation of the metabolism in MCs of *M. hominis* towards the utilisation of deoxyribonucleosides (and ribonucleosides) rather than their production. Downregulation of the enzymes involved in energy-efficient pathways led us to conclusion that the MC state is associated with energy starvation actively induced by *M. hominis* cells. To verify this hypothesis, we constructed and characterised he DeoA-overexpressing strain DeoA:TnRM5. We have shown that the abundance of DeoA protein increased by an average of 4.7 times in strain DeoA:TnRM5 compared with the control strain by the method of differential 2D electrophoresis (Figure S2).

### *deoA* overexpressing mutant reproduces key features of the MC phenotype

**Fig. 4.**
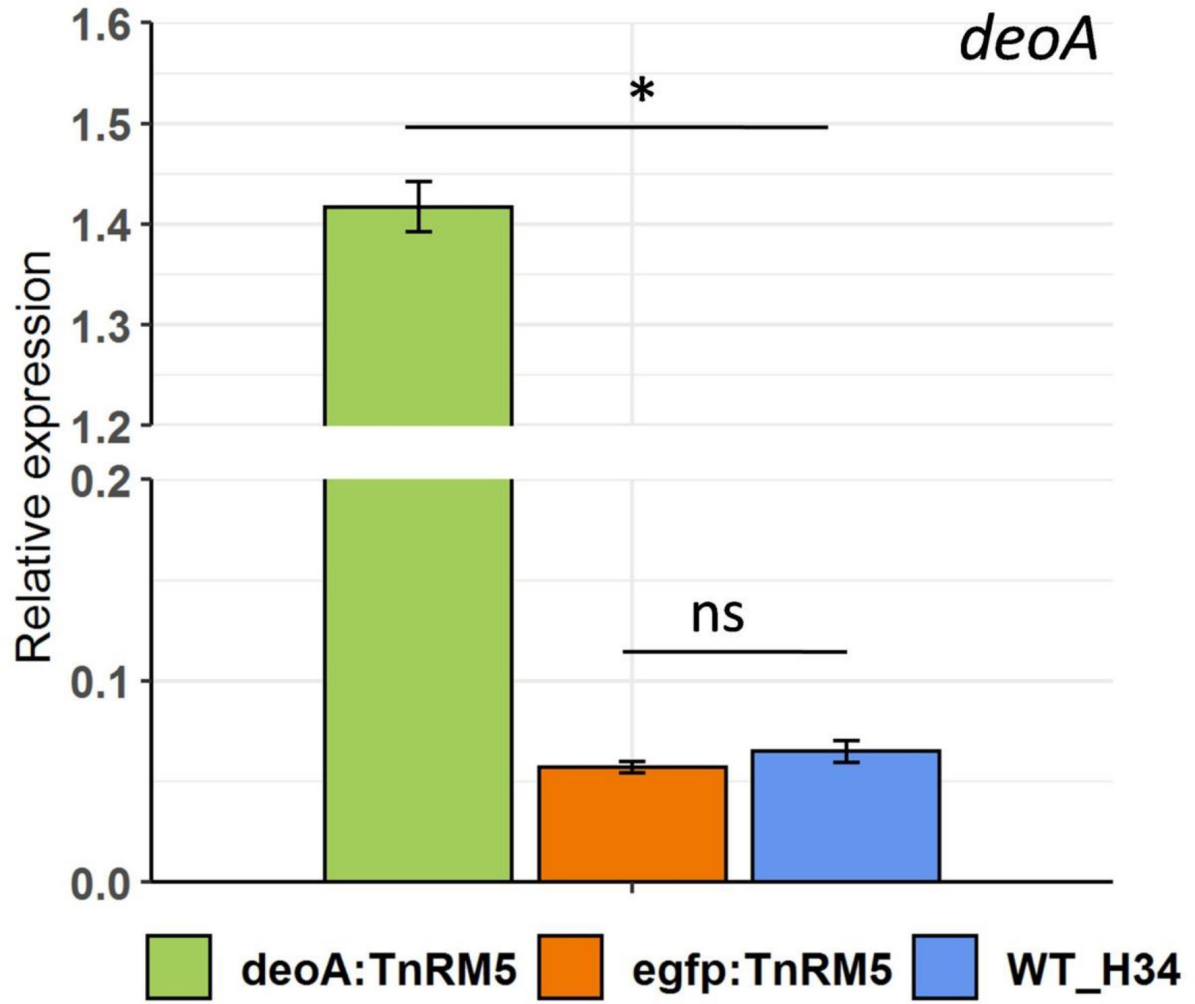
The relative expression levels of *deoA* was determined by qRT-PCR as described in the methods. Each value represents the mean for three experiments, and error bars indicate standard error (*, significantly different from that of the wild type, *P*<0.05; ns, not significant).

The expression level of *deoA* was 21-fold higher in DeoA:TnRM5 strain compared with the WT-H34 strain. The abundance of *deoA* in control EGFP:TnRM5 and WT-H34 strains was equal (Fig. 4). We compared growth rates and the ability to produce MCs in the wild type (WT H-34) and DeoA:TnRM5 bacteria. We have determined that both strains can grow in liquid medium containing thymidine as a carbon source. The growth rate of both WT H-34 and DeoA: TnRM5 was lower on thymidine than on arginine (Fig. 3). The maximum amount of the gDNA was reached in the presence of arginine for WT H-34 at 36 h of growth, in the presence of thymidine for DeoA:TnRM5 after 52 h of growth, and for WT H-34 after 70 h of growth. We did not observe mycoplasma growth without adding a carbon source to the medium during 75 h of cultivation.

**Fig. 5.**
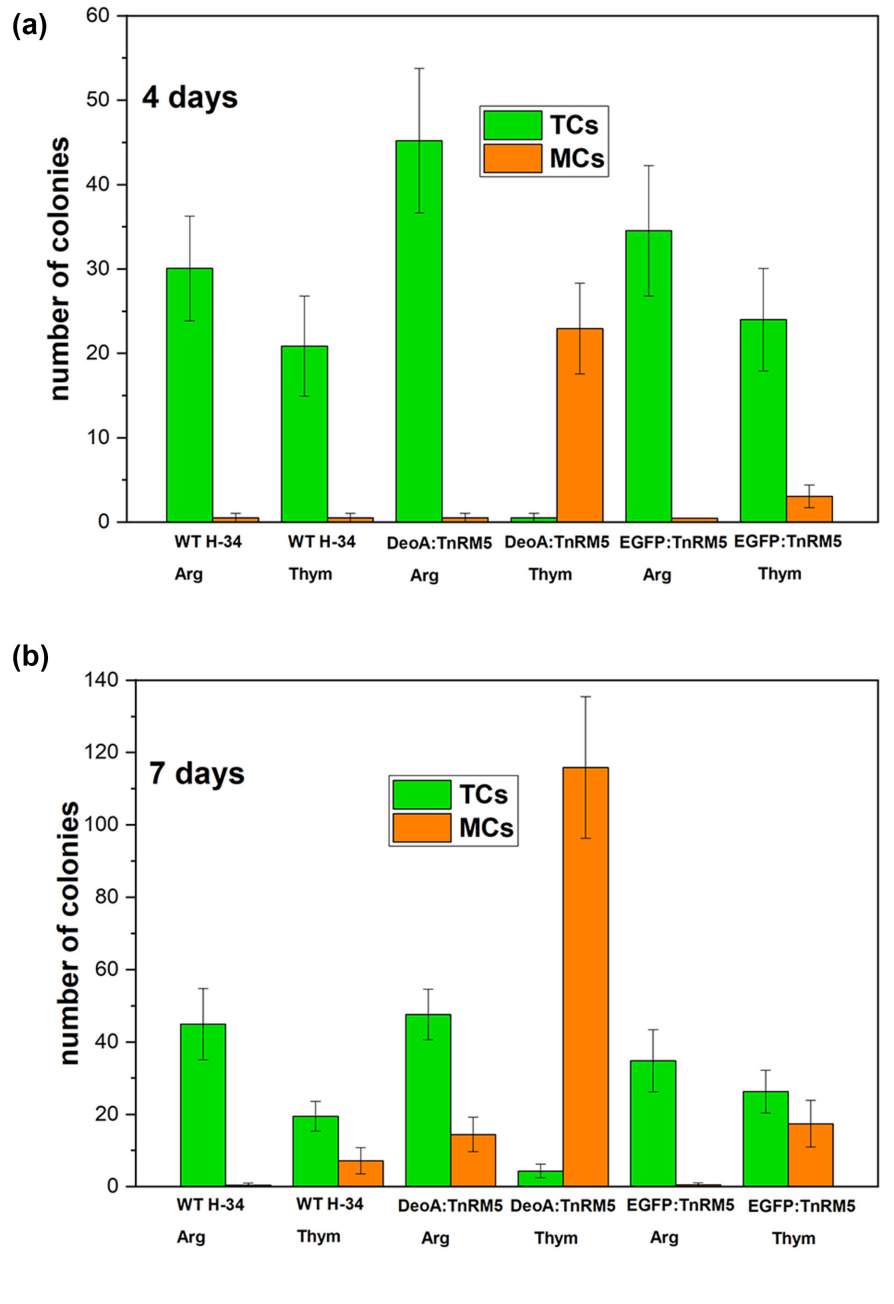
Quantity of TCs and MCs after 4 (**a**) and 7 (**b**) days of cultivation of wild type *M. hominis* H-34 (WT H-34), DeoA:TnRM5 mutant with overexpression of *deoA* gene and EGFP:TnRM5 mutant grown on solid agar supplemented with arginine (WT H-34 Arg, DeoA:TnRM5 Arg, EGFP:TnRM5 Arg) and with thymidine (WT H-34 Thym, DeoA:TnR5 Thym, EGFP:TnRM5 Thym). The number of TCs is indicated in green and MCs in orange. The colonies calculated as the average value in five fields of view on the plate. In each case, three plates were taken for analysis.

**Fig. 6.**
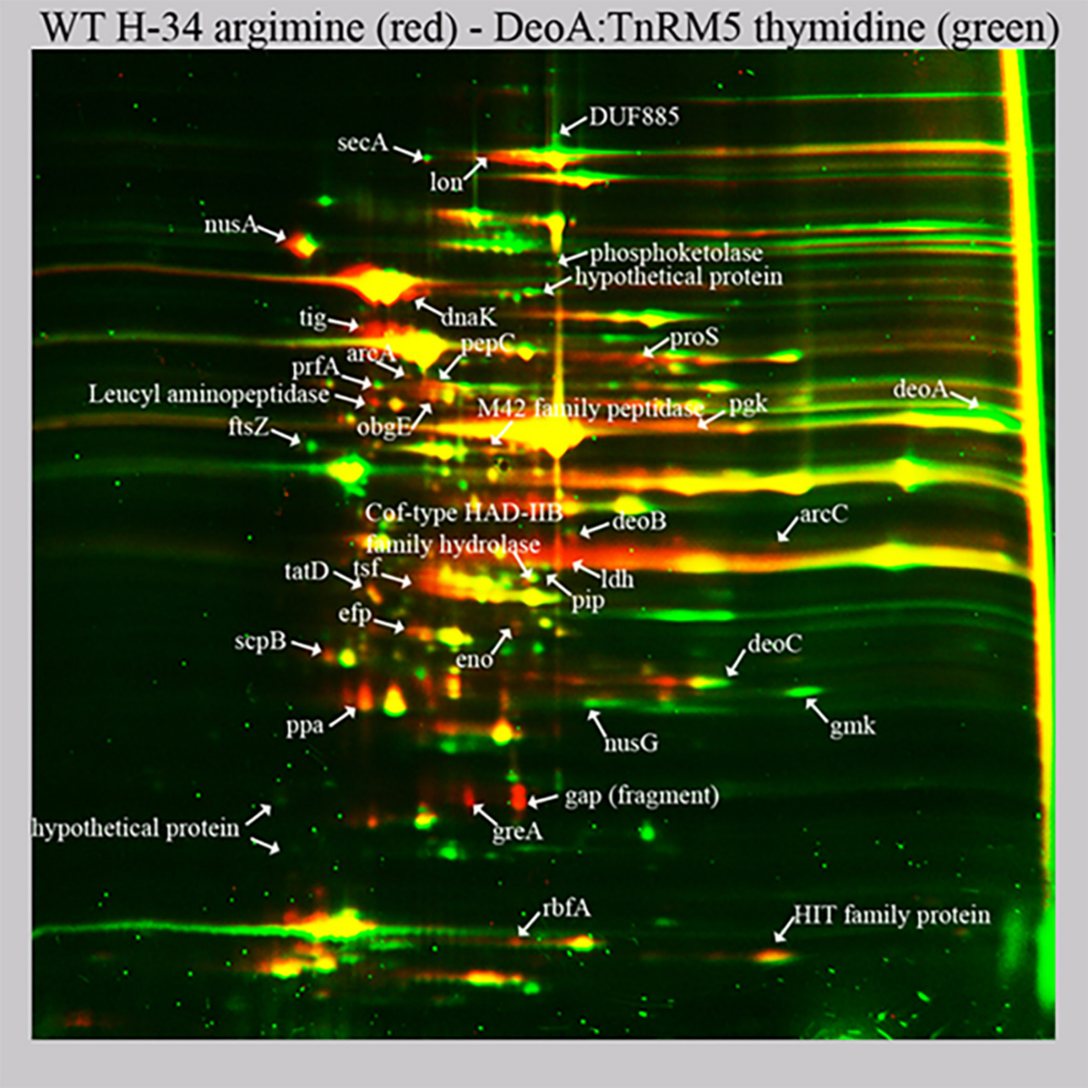
Comparative proteomic analysis of wild type of *M. hominis* H-34 grown on liquid media with arginine (WT H-34 arginine, red spots) and DeoA:TnRM5 mutant grown on liquid media with thymidine (DeoA:TnRM5 thymidine, green spots) revealed by differential 2D gel electrophoresis (pH range 3,5–10). Arrows indicate reliably determined differentially expressed proteins. Protein identification was carried out by a peptide fingerprint search with the use of Mascot software (Matrix Science) through a NCBI protein *M. hominis* ATCC 23114 NCBI database. The cutoff score for protein identification in Mascot engine was 44 (*P*<0.05). The results of quantitative analysis are presented in Table S2.

In order to assess MCs' production we monitored growth of WT strain H-34 and DeoA:TnRM5 on a solid agar. We used the strain with EGFP protein overexpression (EGFP:TnRM5) as a negative control. Expression of *egfp* was confirmed by qRT-PCR (Fig. S3). Strain DeoA:TnRM5 allowed us to exclude the influence of the selectable marker and the transformation procedure on the phenotype of the transformants. The results are shown in Fig. 5. The number of colonies was counted as the average, counted in five fields of view for three agar plates for each condition (Table S3). All strains growing on arginine formed only TCs on day four and MCs became visible on day seven. On day four in thymidine, WT H-34 produced TCs, EGFP:TnRM5 mostly formed TCs with few MCs, and DeoA:TnRM5 only formed MCs and no TCs. On day seven in thymidine, WT H-34 showed few MCs, EGFP:TnRM5 showed near equal number of TCs and MCs and DeoA:TnRM5 predominantly formed MCs, whose number was at least an order of magnitude higher than that of the other strains. Thus, we conclude that DeoA upregulation along with thymidine is essential in the transition of *M. hominis* to the MC phenotype. Furthermore, we used DeoA:TnRM5 cultured on thymidine as a model for transition to the MC state. We performed comparative proteomic analysis of DeoA:TnRM5 on thymidine and WT H-34 on arginine, and compared the changes with those observed in the MCs produced from WT H-34 (Fig. 6, Table S4). Generally, both strains showed the same trends in metabolic pathways; however, the changes in the levels of particular enzymes were not the same (Tables 1 and 2). The most characteristic changes included downregulation of arginine utilisation and upregulation of thymidine catabolism. In MCs, ArcA, ArcB, and DDAH were downregulated while ArcC was upregulated. In DeoA:TnRM5, ArcC was downregulated and DeoA and DeoC were upregulated. At the same time, DeoB was downregulated in both strains. Thus, we conclude that one of the key differences between MCs and TCs is the change in energy metabolism from arginine to thymidine utilisation.

### *deoA* overexpressing mutant and MCs show resistance to antibiotics compared TCs

**Fig. 7.**
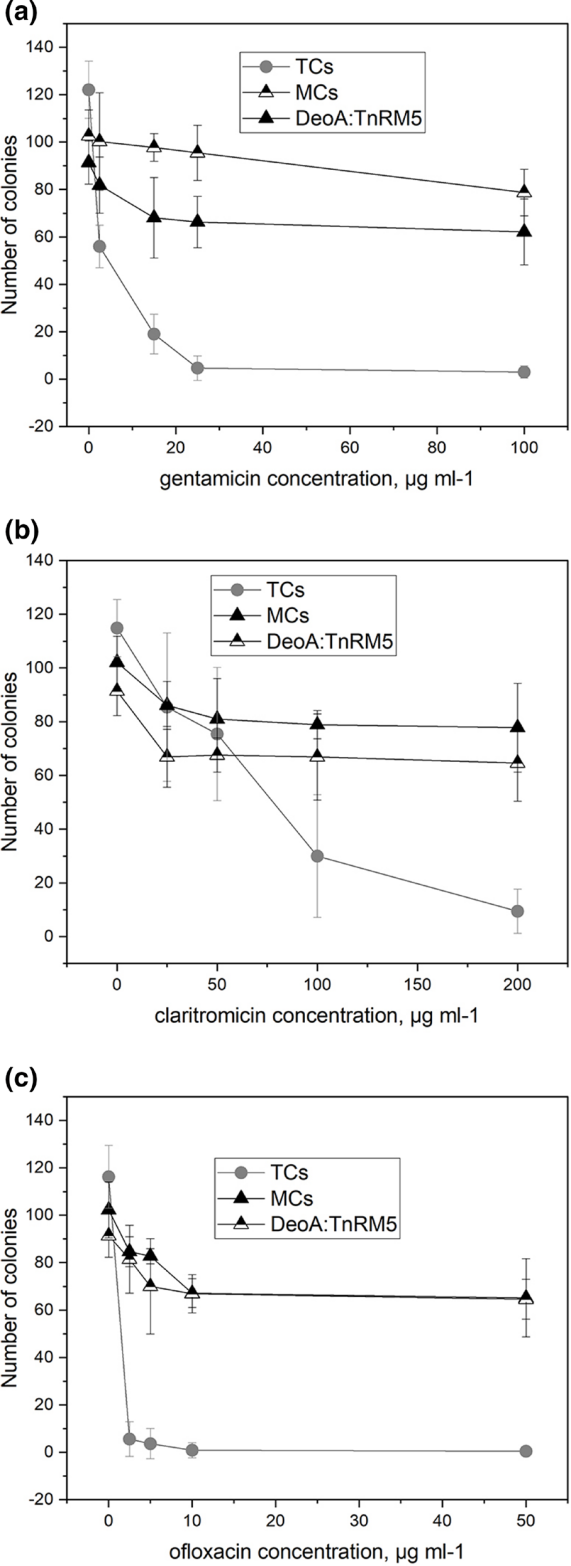
Survival testing of TCs, MCs and DeoA:TnRM5 mutant colonies depending on the concentration of gentamicin (**a**), clarithromycin (**b**) and ofloxacin (**c**) using the agar dilution method. At least three agar plates were used for each antibiotic concentration. The number of colonies was counted as the average, counted in five fields of view in three agar plates using a microscope (LETZLAR).

We assessed resistance of TCs, MCs and the DeoA:TnRM5 mutant to antibiotics (Fig. 7). We used ofloxacin (a fluoroquinolone), gentamicin (an aminoglycoside) and clarithromycin (a macrolide). Ofloxacin inhibits DNA replication by targeting DNA gyrase and topoisomerase IV activity [30]. Gentamicin and clarithromycin block protein synthesis by irreversibly binding to the 16S rRNA of the 30S ribosomal subunit [31] and to the 23S rRNA of the 50S ribosomal subunit respectively [32]. All cultures showed higher resistance to clarithromycin compared with gentamicin and ofloxacin. The survival was higher in the MCs and DeoA:TnRM5 mutant cultures than of TCs on all three antibiotics. Generally an increase in antibiotic concentration led to a decrease in colony number of all cultures. However, the number of colonies of TC culture decreased significantly faster, than those of MC and DeoA:TnRM5 mutant cultures. At certain concentrations of the antibiotics the colony number of MC and DeoA:TnRM5 mutant cultures reached a plateau. For TC culture we observed complete or almost complete growth inhibition. Antibiotic sensitivity testing for DeoA:TnRM5 demonstrated that it was resistant to all three antibiotics, similarly to MCs. These results are consistent with the results of TC and MC antibiotic susceptibility testing using the disc diffusion method [7].

## Discussion

Proteomic analysis of *M. hominis* colonies allowed us to reconstruct their energy metabolism pathway. Until now it was known that *M. hominis* can use glyceraldehyde-3-phosphate generated from the pentose phosphate pathway instead of β-d-glucose in energy metabolism [24]. Moreover, *M. hominis* can utilise arginine to initiate growth [25, 33]. Comparative proteomic analysis of *M. hominis* cultivated on a medium supplemented with arginine or thymidine as a carbon source demonstrated that *M. hominis* can additionally utilise ribose phosphate and deoxyribose phosphate, which are produced by nucleoside catabolism, as an energy source (Fig. 2).

Nucleoside phosphorylases (DeoD and DeoA) are known to catalyse the cleavage of ribonucleosides and deoxyribonucleosides into free bases and ribose-1-phosphate or deoxyribose-1-phosphate, respectively. Nucleoside phosphorylases (DeoD and DeoA) catalyse the reversible cleavage of the glycosidic bond of ribonucleosides or deoxyribonucleosides in the presence of inorganic phosphate [34]. Ribose-1-phosphate can be converted by DeoB to ribose-5-phosphate and subsequently to phosphoribosyl pyrophosphate, which can be used in *de novo* nucleotide synthesis or in glycolysis through the PPP. Similarly, deoxyribose-1-phosphate can be converted by DeoB to deoxyribose-5-phosphate, which, in turn, is converted by the enzyme deoxyriboaldolase to glyceraldehyde-3-phosphate, which is involved in glycolysis. Thus, phosphorylated deoxyribose can only be used in catabolic pathways and cannot be used for nucleotide synthesis.

**Fig. 8.**
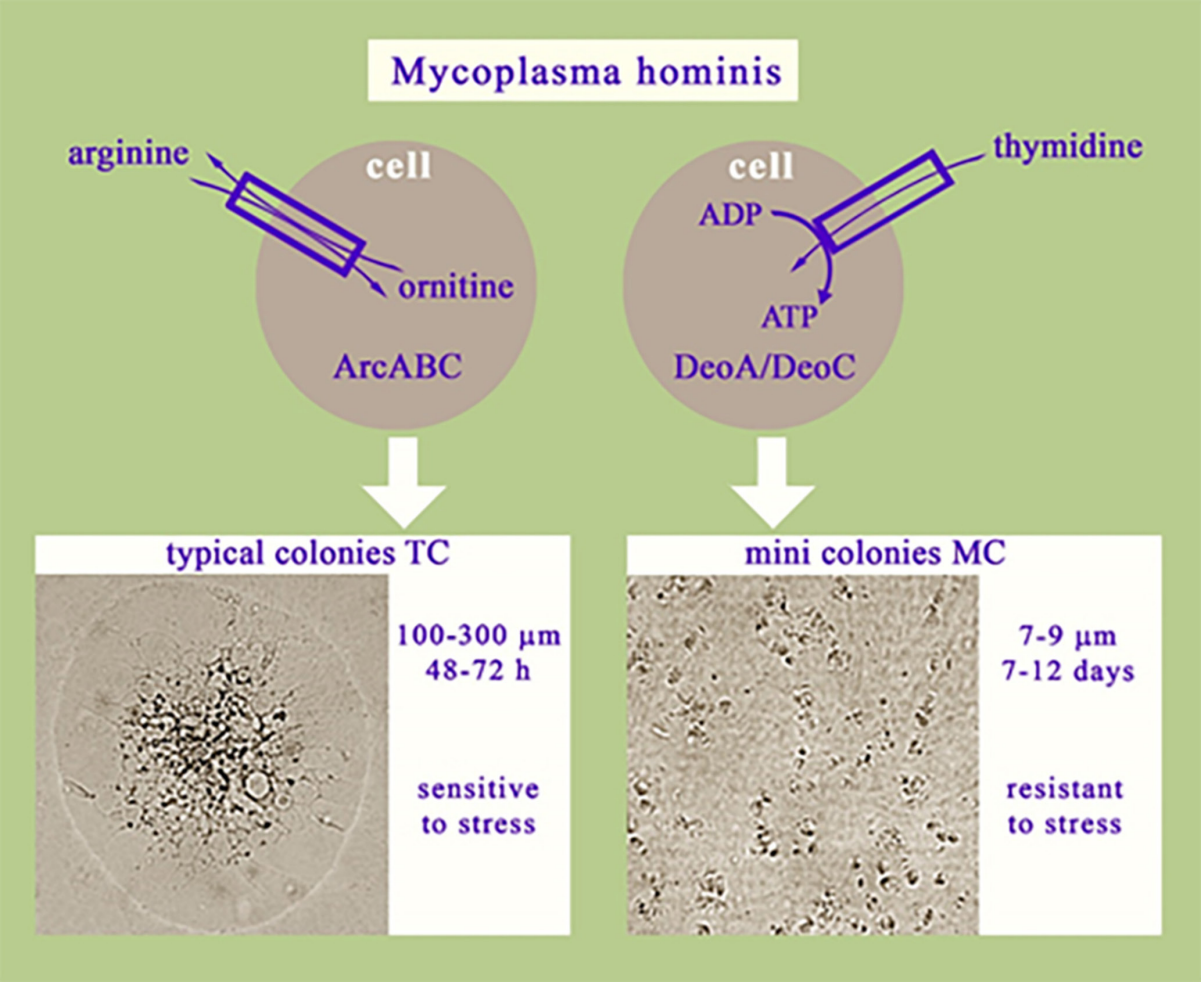
Scheme of metabolic switching in *M. hominis *, leading to the formation of typical colonies (TCs) or mini colonies (MCs).

After comparing the proteomic profiles of TCs and MCs, we observed that MC cells demonstrated a reorganisation of energy metabolism characterised by a decrease in the levels of ArcA and DDAH, key enzymes involved in arginine utilisation, a downregulation of DeoD, which catabolises ribonucleosides, and an upregulation of DeoA, which catabolises deoxyribonucleosides. Therefore, we suggest that MC cells preferably utilise deoxyribonucleosides, particularly thymidine, as an energy source rather than arginine or ribonucleosides (Fig. 8). Our hypothesis was confirmed by a DeoA*-*overexpressing mutant (Deo:TnRM5), which exhibited a similar phenotype to that of MCs.

Utilisation of deoxyribonucleosides is less efficient than that of ribonucleosides in terms of energy production, and the rate of deoxyribonucleoside utilisation is probably lower than that of arginine utilisation. This is also evident from the slow growth of MCs as compared with that of TCs. Thus, we propose that MC cells enter a state of energy starvation, which helps them survive under stress. Acetaldehyde produced during the catabolism of deoxyribonucleotides can also inhibit growth (Fig. 3). A similar cell transition phenomenon was discovered in the 1940s, while studying the mechanism of action of penicillin [35–39]. It has been reported that there is a population of ‘dormant’ cells or persisters, where the main cellular processes are slowed down. Such cells can survive even in the presence of antibiotics. It has also been reported that a low nutrient medium increases the number of persisters in the *E. coli* population [40–42]. The main difference between MCs and classic persisters is the lack of reversion to the actively growing phenotype in the former. It is possible that either the signal required for the transition of MCs to actively dividing cells is currently unknown, or the culture of *M. hominis* is initially heterogeneous and consists of cells that give rise to TCs and MCs and under unfavourable conditions only MC cells survive. The concerted reorganisation of the metabolism of *M. hominis* supports the conclusion that the MC state is tightly regulated. The comparative analysis of genomes and gene expression of TCs and MCs will help us to understand this phenomenon in the future. A study conducted on small colony variants of *Staphylococcus aureus* also reported metabolic changes such as deficiencies in electron transport and thymidine biosynthesis [43]. Small colonies (SC) of *Mycoplasma mycoides* subsp. *mycoides *, a causative agent of bovine pleuropneumonia, have been identified [44]. They are considered one of the most pathogenic among mycoplasmas. SCs are distinguished by colony morphology, poor growth *in vitro*, and a high degree of sensitivity to immune serum [45]. Several determinants of virulence have been identified for them. Among them is the capsular polysaccharide, which appears to be involved in serum resistance to give the body the ability to persist and spread in the host. In addition, there are adhesion factors, immunomodulatory factors and toxic products of the metabolic pathway (in particular, the ability to produce peroxide) [46]. Unlike our MCs, SCs have much larger colony sizes, they are sensitive to treatment with immune serum, their growth does not depend on an energy source and they can grow freely in liquid culture. The authors found no of changes in SC metabolism.

The reorganisation of energetic metabolism in MC is accompanied by a decrease in the abundance of membrane proteins, which mediate host interaction. Mycoplasma lipoproteins perform a variety of functions ranging from nutrient uptake or hydrolysis of substrates to adhesion, virulence, and immunomodulatory activities [47]. In MCs, the abundance of surface membrane proteins (Lmp related protein, P120, P75, LemA, MHO_0730, OppA, OppF and MHO_3200), which play an important role in the pathogenicity of *M. hominis *, decreased (Table S1). Surface proteins P120 and Lmp-related proteins are recognised by the host immune system [48–50]. The surface lipoprotein MHO_0730, which is expressed *in vivo* during natural infections, acts as a nuclease, and is a potent inducer of neutrophil extracellular traps formation (NETosis) [51]. MHO_3200 contains a DUF31 peptidase domain, which is related to the superfamily of trypsin endopeptidases found in various hypothetical proteins and putative lipoproteins in mycoplasmas. MHO_3200 may act as a virulence factor or as a factor facilitating innate immune escape [52]. ABC transporter OppA plays an important role in cytoadhesion [53, 54]. It has been shown that the cytoadhesive substrate binding protein OppA of oligopeptide permease also functions as an ecto-ATPase in *Mycoplasma hominis* [54]. The ecto-ATPase OppA of *M. hominis* is able to induce the release of ATP from the cells of the host organism and hydrolyse ATP, which ultimately leads to the induction of apoptosis [55]. The effect of ATPase activity on the adhesion of *M. hominis* to HeLa cells was confirmed by the use of ATPase inhibitors, which reduced the cytoadhesion of mycoplasma to 50 % [56]. Surface lipoproteins of mycoplasma help in evading the host immune response through mechanisms such as antigenic variation of lipoproteins [57, 58]. In mycoplasmas, a pronounced variation in surface proteins is observed, and this mechanism evades the host immune response often resulting in chronic mycoplasma infections [59]. P120 and P75 are the variable surface antigens. MCs were obtained by treating TCs with hyperimmune serum. Cell treatment with antibodies leads to a change in the repertoire of surface variable lipoproteins [19, 20]. Therefore, significant changes in the abundance of surface membrane proteins in MCs are not surprising. The decrease in the level of functionally important membrane proteins can be the result of slow growth or a regulated strategy that allows cells to reduce their virulence, be invisible to the host’s immune system and persist freely in the host body.

### Limitations of the study

We have not yet been able to carry out a comparative analysis of genomes and gene expression of TCs and MCs, which is necessary to study the mechanism of MC resistance to unfavourable factors, in particular, to antibiotics. We plan to do this analysis in the future.

## Supplementary Data

Supplementary material 1
