# Supplementary material for: Thymidine utilisation pathway is a novel phenotypic switch of Mycoplasma hominis
Source: J Med Microbiol. 2022 Jan 17;71(1):001468. doi: 10.1099/jmm.0.001468 (PMC8895549; doi:10.1099/jmm.0.001468)
Supplement: Supplementary material 1 [file jmm-71-1468-s001.pdf]

Supplementary

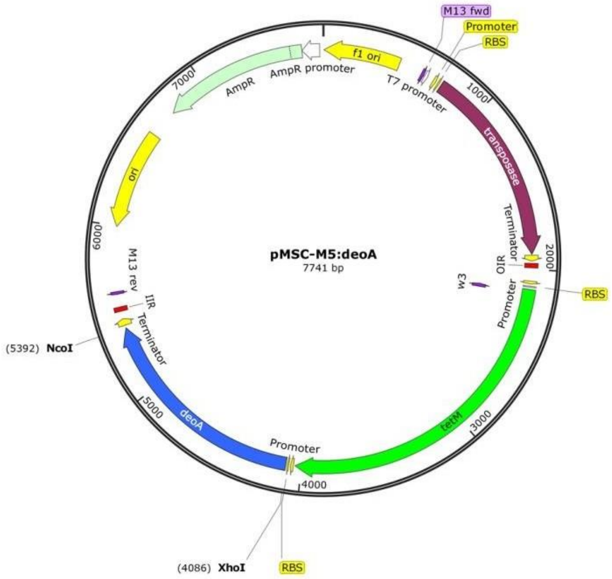

Figure S1. pMSC-M5 transposon vector scheme with constitutive strong promoter and optimal RBS sequence.

Table S1. Primers used for qRT-PCR

Primers	Sequences (5’ - 3’)
Mho_23S-F	CAAATCCGCATAACTTAACTGG
Mho_23S-R	ATCTTACGACTTAGCATAGAGC
Mho_eno-F	CCAAGTAGCATACAAAATGCCA
Mho_eno-R	CTGAAGTAGCTGGATTATATCCTG
Mho_tuf-F	TATTGCTACGTGGAATTGACAG
Mho_tuf-R	CCTTCACGAATAGAGAACTTGG
Mho_deoA-F	TGATAAACACTCAACAGGAGGA
Mho_deoA-R	CAATGGAATTGATTGAACGGTA
Egfp-F	GACCCTGAAGTTCATCTGCACC
Egfp-R	TAGTTGTACTCCAGCTTGTGCC

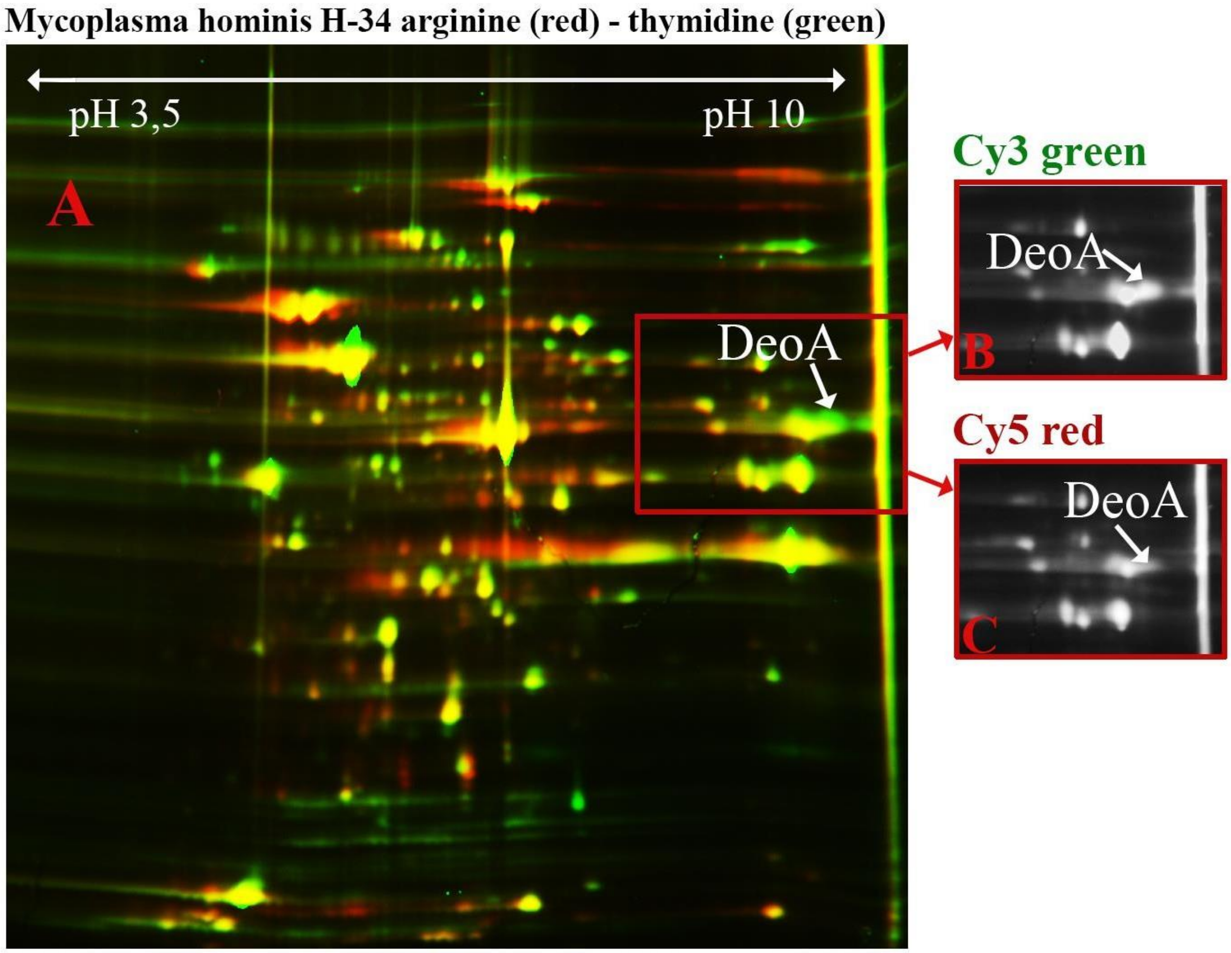

Figure S2. Comparative proteomic analysis of wild type of *M. hominis* H-34grown on liquid media with arginine (WT H-34arginine, red spots) and thymidine (WT H-34, thymidine,green spots) revealed by differential 2D gel electrophoresis (pH range 3,5–10). Arrows indicate reliably determined differential protein DeoA. Protein identification was carried out by a peptide fingerprint search with the use of Mascot software (Matrix Science Inc., USA) through a NCBI protein *M. hominis* ATCC 23114 NCBI database. The cutoff score for protein identification in Mascot engine was 44 ($p<0.05$). The insets show fragment of 2D gel (A), scanned separately at wavelengths 532 nm (B, Cy3, green) and 633 nm (C, Cy5, red).

Table S2. Changes of the proteins in MCs compared with TCs for three biological replicates – OP1, OP2 and OP3 calculated using LC-MS analysis and label-free identification with MaxQuant 1.6.6.0.

ID	L2FC_OP1 MCs/TCs	L2FC_OP2 MCs/TCs	L2FC_OP3 MCs/TCs	Gene	Protein
D1J8H2	-0.94163	NA	-0.29431	ackA	Acetate kinase
D1J7R8	-0.31076	-0.034842	-0.48369	alaS	Alanine--tRNA ligase
D1J7K6	-1.54548	-0.826487	-1.54274	arcA	Arginine deiminase
D1J7K1	-0.24871	0.3552832	-0.0807	arcB	Ornithine carbamoyltransferase
D1J7K0	0.63613	0.3768503	-0.42056	arcC	Carbamate kinase
D1J7J7	0.49064	-0.6719	-0.57001	argS	Arginine--tRNA ligase
D1J8F3	-0.23339	30	-1.17836	asnA	Aspartate--ammonia ligase
D1J8N7	-0.05987	1.0249757	1.707126	asnS	Asparagine--tRNA ligase
D1J7G4	-0.37572	-1.188635	-1.34401	atpA	ATP synthase subunit alpha
D1J8A1	-0.85968	-0.048223	-30	atpA	ATP synthase alpha chain
D1J8A0	-0.80103	-30	-1.13743	atpD	ATP synthase beta chain
D1J7G6	-1.36794	NA	-1.41451	atpD	ATP synthase subunit beta
D1J7G2	-1.60241	-30	-30	atpF	ATP synthase subunit b
D1J7G3	-2.47185	-30	NA	atpH	ATP synthase subunit delta
D1J7Y4	-1.33417	-0.036404	0.287506	cbiO1	Energy-coupling factor transporter ATP-binding protein EcfA
D1J7Y5	-1.52534	-30	-0.70701	cbiO2	Energy-coupling factor transporter ATP-binding protein EcfA
D1J845	-0.39246	-30	-0.35394	cdd	Cytidine deaminase
D1J7V4	-0.11084	NA	0.58641	cmk	Cytidylate kinase
D1J8C2	0.7585	0.629534	3.388946	deoA	Thymidine phosphorylase
D1J8K5	-0.80145	-1.854743	-0.88987	deoB	Phosphopentomutase
D1J8C1	0.00268	30	-0.12523	deoC	Deoxyribose-phosphate aldolase
D1J8C3	-1.49061	0.1770188	-0.51067	deoD	Purine-nucleoside phosphorylase
D1J7Y0	-0.15653	30	0.074937	dgk	Deoxyguanosine kinase
D1J7E1	-1.48627	30	-0.2009	dnaA	Chromosomal replication initiator protein DnaA
D1J8H9	-0.58648	NA	-0.311	dnaB	Replicative DNA helicase
D1J8K0	0.13498	NA	-0.38765	dnaE	DNA-directed DNA polymerase
D1J803	-0.38755	NA	-0.44223	dnaG	DNA primase
D1J7U9	-1.27326	NA	-30	dnaJ	Chaperone protein DnaJ
D1J8T2	-0.44679	0.5317395	-0.90177	dnaK	Chaperone protein DnaK
D1J7E2	0.83205	1.0415974	-3.10609	dnaN	DNA polymerase III beta chain
D1J7N2	-0.50249	30	-2.06112	dnaX	DNA polymerase III subunit gamma/tau
D1J7V5	-0.52409	-1.20346	-1.23778	engA	GTPase Der (GTP-binding protein EngA)
D1J8I2	-1.04229	NA	-30	engB	Probable GTP-binding protein EngB
D1J7M6	-0.2083	-30	0.191144	eno	Enolase

D1J8N1	0.0965	30	-0.22532	era	GTPase Era
D1J7K4	-1.0192	NA	-30	fba	Fructose-bisphosphate aldolase
D1J805	-1.48485	30	-2.24434	ffh	Signal recognition particle protein
D1J8M2	0.49069	-0.686885	-1.45733	fmt	Methionyl-tRNA formyltransferase
D1J8L1	-1.44158	-0.065977	0.322371	ftsH	ATP-dependent zinc metalloprotease FtsH
O32861	-0.14282	30	-30	ftsY	Signal recognition particle receptor FtsY
D1J814	-0.46264	-0.643002	-0.17299	ftsZ	Cell division protein FtsZ
D1J7T4	-0.25764	1.4673069	-0.64714	fusA	Elongation factor G
D1J8V2	-0.44481	2.063017	-0.48762	gap	Glyceraldehyde-3-phosphate dehydrogenase
D1J8E2	-0.60507	-1.44609	NA	gidB	Ribosomal RNA small subunit methyltransferase G
D1J8S4	0.39837	30	-0.23759	gmk	Guanylate kinase
D1J7V6	-1.27583	30	1.130694	gpsA	Glycerol-3-phosphate dehydrogenase
D1J7S2	0.35352	0.5217446	7.996368	greA	Transcription elongation factor GreA
D1J8J6	0.24449	30	-0.58289	grpE	Protein GrpE
D1J8L4	-1.32893	1.8910299	-0.55494	gyrA	DNA gyrase subunit A
P43053	-1.09547	-1.635023	-0.9404	gyrB	DNA gyrase subunit B
D1J7Y1	-1.11525	NA	-0.9246	hpt	Hypoxanthine phosphoribosyltransferase
D1J8J7	-1.34008	30	-1.78894	hrcA	Heat-inducible transcription repressor HrcA
D1J8V8	0.0572	30	-2.54867	hsdM	Type I restriction enzyme M protein
D1J8C9	-1.28886	NA	0.737745	lgt	Phosphatidylglycerol--prolipoprotein diacylglyceryl transferase
D1J893	0.06294	-30	-0.7263	ileS	Isoleucine--tRNA ligase
D1J8E6	0.27753	-0.368376	-0.50651	ldh	L-lactate dehydrogenase
P43054	-1.31919	0.778767	0.584496	lemA	Protein LemA
D1J8E8	-2.24966	-30	NA	rpsA	30S ribosomal protein S1
D1J7Y7	0.59586	NA	30	ligA	DNA ligase
D1J7J0	-1.09926	-30	1.0394	lmp1	Lmp1 protein
D1J7V1	3.52637	NA	NA	lmp3	Lmp3 protein
D1J8J5	-0.11234	NA	-0.73513	lon	Lon protease
P46191	-0.07383	NA	-0.3934	lysS	Lysine--tRNA ligase
D1J8F2	1.94391	NA	3.394012	metK	S-adenosylmethionine synthase (AdoMet synthase
D1J8E4	-1.32714	-2.399082	-0.03959	mgtA	Cation-transporting P-ATPase
D1J7D8	-1.00769	30	-0.30747	MHO_0010	Uncharacterized protein
D1J7E6	-1.09019	NA	-30	MHO_0090	Uncharacterized protein
D1J7F1	3.48971	NA	5.87622	MHO_0140	Pseudogen of Type III restriction modification system: methylase (Part 1)
D1J7G8	-2.36868	NA	-1.00119	MHO_0280	Uncharacterized protein
D1J7G9	-2.12493	30	-1.00119	MHO_0290	Uncharacterized protein
D1J7H2	-1.14693	NA	-0.28459	MHO_0320	Lipase_GDSL domain-containing protein
D1J7I8	-1.37287	30	-30	MHO_0510	Diadenylate cyclase

D1J7K3	-1.16519	-30	-3.11907	MHO_0660	TNase-like domain-containing protein
D1J7K8	-1.40798	-30	0.222646	MHO_0710	Uncharacterized protein
D1J7K9	-1.15808	2.0399397	0.901646	MHO_0720	Uncharacterized protein
D1J7L1	-0.94748	-0.221746	-0.4607	MHO_0740	ABC transporter ATP-binding protein
D1J7L5	-1.53713	NA	-30	MHO_0780	Uncharacterized protein
D1J7L6	-1.31169	1.2415763	-0.09199	MHO_0790	Uncharacterized protein
D1J7M7	-0.46358	NA	-30	MHO_0900	Uncharacterized protein
D1J7N0	-1.79345	-30	-30	MHO_0930	Diadenosine 5'5'''-P1, P4-tetraphosphatepyrophosphohydrolase
D1J7P5	-1.0356	NA	-1.87975	MHO_1080	ATP-binding protein
D1J7P6	-1.92343	NA	-30	MHO_1090	Uncharacterized protein
D1J7Q3	-0.58132	-30	30	MHO_1160	ATPase, AAA family
D1J7Q7	-0.05191	-30	-2.6317	MHO_1200	Uncharacterized protein
D1J7R9	-1.44139	-0.121768	-0.20006	MHO_1320	Putative pre-16S rRNA nuclease
D1J7S1	-0.6678	NA	-3.53988	MHO_1340	Uncharacterized protein
D1J7S3	-0.8185	-30	-30	MHO_1360	DNA methylase
D1J7S9	-0.639	-30	1.225794	MHO_1420	Uncharacterized protein
D1J7T0	-1.78334	1.0950685	-0.12699	MHO_1430	Uncharacterized protein
D1J7T6	-0.53478	NA	-30	MHO_1490	Uncharacterized protein
D1J7T7	-0.30912	-2.688212	NA	MHO_1500	Deoxycytidylate deaminase
D1J7V2	-0.97556	NA	-30	MHO_1650	Uncharacterized protein
D1J7V8	-2.6367	NA	-30	MHO_1710	DUF2779 domain-containing protein
D1J7V9	-1.10884	0.1729754	-30	MHO_1720	Uncharacterized protein
D1J7W0	-0.64593	-30	-30	MHO_1730	Uncharacterized protein
D1J7W3	-1.61979	-0.906074	NA	MHO_1760	Iron-containing alcohol dehydrogenase
D1J7Z5	-0.67756	30	NA	MHO_2080	TSPc domain-containing protein
D1J806	-0.58552	30	-30	MHO_2190	Lipase_GDSL domain-containing protein
D1J807	-0.55467	NA	3.867708	MHO_2200	Ribosome biogenesis GTPase A
D1J812	-1.19442	NA	-30	MHO_2250	Ribosomal RNA small subunit methyltransferase E
D1J815	-0.54307	-30	-30	MHO_2280	Uncharacterized protein
D1J818	-1.87736	-30	NA	MHO_2310	ABC transporter, ATP-binding protein
D1J819	-2.20037	NA	-30	MHO_2320	ABC transporter, ATP-binding protein
D1J823	-0.38005	NA	-1.72553	MHO_2360	Uncharacterized protein
D1J831	-1.438	NA	2.475466	MHO_2440	Uncharacterized protein
D1J841	-1.00122	-30	-1.07731	MHO_2540	N-dimethylarginine dimethylaminohydrolase
D1J844	-0.93174	NA	-30	MHO_2570	HTH cro/C1-type domain-containing protein
D1J851	0.00065	NA	0.097853	MHO_2640	Uncharacterized protein
D1J889	-0.38961	-1.057646	-1.22949	MHO_3010	Phosphoketolase
D1J895	-1.29114	NA	-0.32337	MHO_3070	Lmp related protein
D1J898	-0.51176	NA	-30	MHO_3100	p75 related protein, predicted lipoprotein
D1J8A2	-1.4724	NA	-4.10431	MHO_3140	Uncharacterized protein

D1J8A4	-1.37267	-30	-30	MHO_3160	Uncharacterized protein
D1J8A7	-0.95283	NA	-0.88993	MHO_3190	Uncharacterized protein
D1J8A8	-1.75672	-1.488317	-0.7316	MHO_3200	DUF31 domain-containing protein
D1J8B9	0.00774	30	-0.08297	MHO_3310	Uncharacterized protein
D1J8C6	-0.18535	-0.252512	-0.18885	MHO_3380	Lactamase_B domain-containing protein
D1J8D2	-0.13127	30	-0.64335	MHO_3440	Uncharacterized protein
D1J8D3	-0.56994	NA	0.139495	MHO_3450	Uncharacterized protein
D1J8D7	-0.32654	0.0881009	-0.42422	MHO_3490	Membrane protein P60
D1J8D8	-1.22275	0.4008253	-0.44228	MHO_3500	Membrane protein P80
D1J8E1	-1.37185	-30	0.731355	MHO_3530	ABC transporter permease protein
D1J8E9	-1.67732	-30	NA	MHO_3610	p37-like ABC transportersubstrate-binding lipoprotein
D1J8F0	-1.59297	0.5831355	-0.21065	MHO_3620	p37-like ABC transportersubstrate-binding lipoprotein
D1J8G1	-1.17805	-30	-30	MHO_3730	Lmp related protein
P43055	-1.41816	NA	-1.11617	MHO_3780	Uncharacterized protein
D1J8I6	-0.66509	30	-0.17401	MHO_3980	Endo-1,4-beta-glucanase
D1J8K2	-1.1798	-30	-0.27491	MHO_4140	Uncharacterized protein
D1J8L6	-1.26824	NA	-30	MHO_4280	Lmp related protein
D1J8M3	-1.17746	NA	-1.16472	MHO_4350	ABC2_membrane domain-containing protein
D1J8M8	-1.10308	30	-0.31803	MHO_4400	Uncharacterized protein
D1J8N2	-0.44929	NA	-0.20479	MHO_4440	Uncharacterized protein
D1J8N6	-0.58304	-0.766483	-1.39281	MHO_4480	Uncharacterized protein
D1J8N8	-1.46326	NA	-1.46408	MHO_4500	Glycosyltransferase
D1J8P1	-2.94606	NA	-0.41358	MHO_4530	Uncharacterized protein
D1J8P5	-1.61598	-0.804265	30	MHO_4570	TRAM domain-containing protein
D1J8Q5	-0.64283	30	-0.5439	MHO_4670	DhaL domain-containing protein
D1J8R0	-1.44199	30	-1.30367	MHO_4720	Uncharacterized protein
D1J8U2	-1.17416	1.3682603	0.439263	MHO_5040	Uncharacterized protein
D1J8U7	-0.91925	0.7933394	-1.45169	MHO_5090	Xaa-His dipeptidase
D1J8V7	-0.65205	-2.065426	-30	MHO_5200	S1 motif domain-containing protein
D1J8W8	-0.29187	0.8241865	-0.46088	MHO_5310	Uncharacterized protein
D1J8W9	-0.63252	1.3511386	-0.49285	MHO_5320	Uncharacterized protein
D1J8X1	-0.51889	-0.434825	-30	MHO_5340	Nicotinate phosphoribosyltransferase
D1J816	-1.39714	30	-0.839	mraW	Ribosomal RNA small subunit methyltransferase H
D1J817	-2.36221	-30	4.068585	mraZ	Transcriptional regulator MraZ
D1J8H4	-1.30739	-0.92227	2.482687	mutM	Formamidopyrimidine-DNA glycosylase
D1J7R3	-0.43917	-3.053665	0.593296	nadD	Probable nicotinate-nucleotide adenylyltransferase
D1J8W2	0.11908	NA	-1.45908	nusA	Transcription termination/antitermination protein

D1J8I7	-1.01441	30	-1.61506	nusG	Transcription termination/antitermination protein
D1J7S5	-0.87225	-0.980654	-0.61207	obg	GTPase Obg
D1J7T8	-1.52222	-0.345991	-0.42255	oppA	Oligopeptide ABC transporter substrate-binding protein
D1J7U0	-1.0796	-0.927441	NA	oppC	Oligopeptide transport system permease protein
D1J7W2	0.67118	NA	1.282819	oppC	Oligopeptide transport system permease protein
D1J7U1	-1.79687	30	-0.30929	oppD	Oligopeptide transport ATP-binding proteinhomolog
D1J7U2	-1.97706	-0.618394	-0.5292	oppF	Oligopeptide transport ATP-binding proteinhomolog
D1J8F4	-1.6026	-0.970873	0.151669	p120	p120
D1J8G8	-1.76089	0.2387613	0.045726	p120'	p120' protein
D1J8V0	-0.64771	-30	-0.96351	parE	DNA topoisomerase
D1J8L8	-0.72354	1.9287605	-0.61763	pepA	Aminopeptidase
D1J8L9	-0.24872	1.2767693	-30	pepA	Aminopeptidase
D1J7H5	-0.02725	-0.137028	-0.93345	pepC	Aminopeptidase
D1J8P2	-0.79818	0.9833418	-2.15044	pepO	Endopeptidase O
D1J7Z9	-1.57944	-0.575029	-0.53659	pepQ	XAA-Pro dipeptidase
D1J8E7	-0.52137	-30	-0.86634	pgi	Glucose-6-phosphate isomerase
D1J7I6	0.28995	-30	-1.22556	pgk	Phosphoglycerate kinase
D1J8E5	-0.87813	-30	-30	phnC	Phosphonates import ATP-binding protein
D1J7H9	-0.97987	-1.969566	-0.53305	plsC	1-acyl-SN-glycerol-3-phosphate acyltransferase
D1J8K1	0.00277	-2.710038	-1.40973	polA	DNA polymerase I
D1J8Q1	-0.43044	-30	-0.45412	polC	DNA polymerase III PolC-type
D1J8N3	-1.68954	0.3875989	-2.45805	potA	Spermidine/putrescine ABC transporterATP-binding protein potA
D1J7X0	-0.35411	-0.237978	-30	ppa	Inorganic pyrophosphatase
D1J8P9	0.58027	-0.590499	NA	frr	Ribosome recycling factor
D1J8E3	-0.66168	0.4161066	-0.41262	prs	Ribose-phosphate pyrophosphokinase
D1J8K6	-0.36702	-0.717101	-1.21193	pyk	Pyruvate kinase
D1J8P8	-0.44721	-30	-0.26606	pyrH	Uridylate kinase
D1J7Q6	1.56651	-0.199818	-0.16667	recA	Protein RecA
D1J7I2	-1.3253	NA	-1.53252	rluC	Ribosomal large subunit pseudouridine synthaseC
D1J856	0.58745	0.1536693	NA	rplL	50S ribosomal protein L7/L12
D1J7M5	-0.47514	-0.709331	-0.26884	rplA	50S ribosomal protein L1
D1J810	-0.46765	-0.788058	30	rpsI	30S ribosomal protein S9
D1J7F3	-1.20204	-30	-1.12292	gatA	Glutamyl-tRNA(Gln)amidotransferase subunit A
D1J804	-1.24199	-30	-1.78652	glyQS	Glycyl-tRNA synthetase
D1J874	-1.41932	-0.19698	0.409159	rplE	50S ribosomal protein L5
D1J811	-1.312	NA	-0.2017	rplM	50S ribosomal protein L13
D1J879	-2.88911	1.3737136	-0.98119	rplP	50S ribosomal protein L16

D1J870	-1.28205	-30	-0.88617	rplR	50S ribosomal protein L18
D1J848	-1.52238	-1.241121	-0.19541	rplS	50S ribosomal protein L19
D1J7R6	-1.52949	NA	0.437341	rplT	50S ribosomal protein L20
Q8GM55	-1.45787	-30	0.14232	rplV	50S ribosomal protein L22
D1J884	-1.08179	-1.597273	0.687914	rplW	50S ribosomal protein L23
D1J875	-1.80433	-2.77331	-0.92832	rplX	50S ribosomal protein L24
D1J880	-1.13738	0.364156	-30	rpsC	30S ribosomal protein S3
D1J7L7	-1.52914	-3.807663	0.149237	rpsD	30S ribosomal protein S4
D1J869	-1.55456	-1.246903	-0.06931	rpsE	30S ribosomal protein S5
D1J7P9	-2.01584	-0.553604	-30	rpsF	30S ribosomal protein S6
D1J7T3	-1.08826	-30	-0.32256	rpsG	30S ribosomal protein S7
D1J872	-1.46347	-2.143434	-0.36536	rpsH	30S ribosomal protein S8
D1J861	-2.00937	30	-3.42444	rpsK	30S ribosomal protein S11
D1J860	-0.9704	-0.880776	-0.96002	rpoA	DNA-directed RNA polymerase subunit alpha
D1J855	-0.92746	0.8616815	-1.43831	rpoB	DNA-directed RNA polymerase subunit beta
D1J854	-1.12722	-0.291367	-0.69278	rpoC	DNA-directed RNA polymerase subunit beta'
D1J802	-1.33558	0.6407291	-0.68659	rpoD	RNA polymerase sigma factor rpoD
D1J877	-1.17645	NA	-0.93581	rpsQ	30S ribosomal protein S17
D1J7Q1	-1.00698	-0.428693	-0.41965	rpsR	30S ribosomal protein S18
D1J8P4	-1.11066	NA	-0.57833	trme	tRNA modification GTPase MnmE
D1J7P7	-0.96184	-0.398067	0.574451	def	Peptide deformylase
D1J8W4	0.02115	0.1738247	-0.54516	infB	Translation initiation factor IF-2
D1J894	-0.58592	1.0256649	-0.95192	leuS	Leucine--tRNA ligase
D1J8P3	-0.45576	NA	-1.48646	MHO_4550	tRNA(Met) cytidine acetate ligase
D1J8W6	-0.99503	0.058316	-0.10851	mnmG	tRNA uridine 5-carboxymethylaminomethyl modification enzyme MnmG
D1J7Q4	-0.93838	30	-0.39109	pheS	Phenylalanine--tRNA ligase alpha subunit
D1J7Q5	-0.53079	-0.355784	-1.41866	pheT	Phenylalanine--tRNA ligase beta subunit
Q8GM57	-0.78	-1.463005	-0.40872	rplB	50S ribosomal protein L2
D1J886	-0.28464	-0.232891	-0.34033	rplC	50S ribosomal protein L3
D1J885	-0.90575	-0.191898	-0.33116	rplD	50S ribosomal protein L4
D1J8Q8	-0.28631	-0.707207	-0.50427	smc	Chromosome partition protein Smc
D1J7Q0	-0.84103	-1.046816	NA	ssb	Single-stranded DNA-binding protein
D1J8J1	-1.27698	-30	-0.85732	thiI	Probable tRNA sulfurtransferase
D1J857	-0.27204	30	0.11686	rplJ	50S ribosomal protein L10
D1J7M8	0.10386	-0.207632	-0.40269	tig	Trigger factor
D1J7W4	-0.27835	NA	0.792189	tktA	Transketolase
D1J7Q9	-2.28194	NA	-0.33536	tlyA	Hemolysin A
D1J827	-30	-30	-30	tpiA	Triosephosphate isomerase
D1J876	-0.49777	-0.571293	-1.60829	rplN	50S ribosomal protein L14
D1J8T8	-0.30868	2.7771294	-0.86587	rpsB	30S ribosomal protein S2
D1J887	-0.73901	-2.202079	-0.73938	rpsJ	30S ribosomal protein S10

D1J7T2	-0.29897	-1.005016	-30	rpsL	30S ribosomal protein S12
P22679	0.13094	0.0856585	-0.00204	tuf	Elongation factor Tu
D1J8D0	-1.64797	30	-4.27181	uvrA	UvrABC system protein A
D1J7I5	-0.93708	-0.468567	0.087314	valS	Valine--tRNA ligase
D1J7J3	-1.39899	-1.653187	-30	ychF	Ribosome-binding ATPase

Table S3. Adjusted P-values for pairwise comparison of all groups in each experiment

	TCs Thym DeoA:TnRM5	MCs Thym DeoA:TnRM5	TCs Arg DeoA:TnRM5	MCs Arg DeoA:TnRM5	TCs Thym WT H-34	MCs Thym WT H-34	TCs Arg WT H-34	MCs Arg WT H-34	TCs Thym EGFP:TnRM5	MCs Thym EGFP:TnRM5	TCs Arg EGFP:TnRM5
**MCs DeoA:TnRM5 Thym**	**3.99E-12**	-	-	-		-	-	-	-	-	-
**TCs DeoA:TnRM5 Arg**	**1.38E-13**	**5.01E-10**	-	-	-	-	-	-	-	-	-
**MCs DeoA:TnRM5 Arg**	**5.65E-07**	**3.95E-12**	**1.55E-12**	-	-	-	-	-	-	-	-
**TCs WT H-34 Thym**	**1.21E-10**	**9.03E-12**	**2.90E-13**	**9.95E-11**	-	-	-	-	-	-	-
**MCs WT H-34 Thym**	**0.146**	**3.95E-12**	**2.17E-15**	**5.26E-06**	**2.50E-10**	-	-	-	-	-	-
**TCs WT H-34 Arg**	**2.03E-10**	**3.96E-11**	**0.333**	**1.23E-10**	**2.89E-08**	**1.23E-10**	-	-	-	-	-
**MCs WT H-34 Arg**	**5.65E-07**	**3.95E-12**	**1.55E-12**	**NA**	**9.95E-11**	**5.26E-06**	**1.23E-10**	-	-	-	-
**TCs EGFP:TnRM5 Thym**	**1.49E-07**	**2.89E-12**	**1.25E-11**	**1.70E-08**	**0.594**	**3.10E-07**	**4.41E-08**	**1.70E-08**	-	-	-
**MCs EGFP:TnRM5 Thym**	**3.96E-11**	**1.53E-11**	**3.99E-12**	**2.42E-10**	**0.021**	**1.31E-11**	**3.11E-07**	**2.42E-10**	**0.228**	-	-
**TCs EGFP:TnRM5 Arg**	**1.18E-09**	**5.72E-12**	**1.25E-11**	**4.46E-10**	**0.04**	**1.09E-09**	**3.11E-07**	**4.46E-10**	**0.226**	**0.841**	-
**MCs EGFP:TnRM5 Arg**	**5.65E-07**	**3.95E-12**	**1.55E-12**	**NA**	**9.95E-11**	**5.26E-06**	**1.23E-10**	**NA**	**1.70E-08**	**2.42E-10**	**4.46E-10**

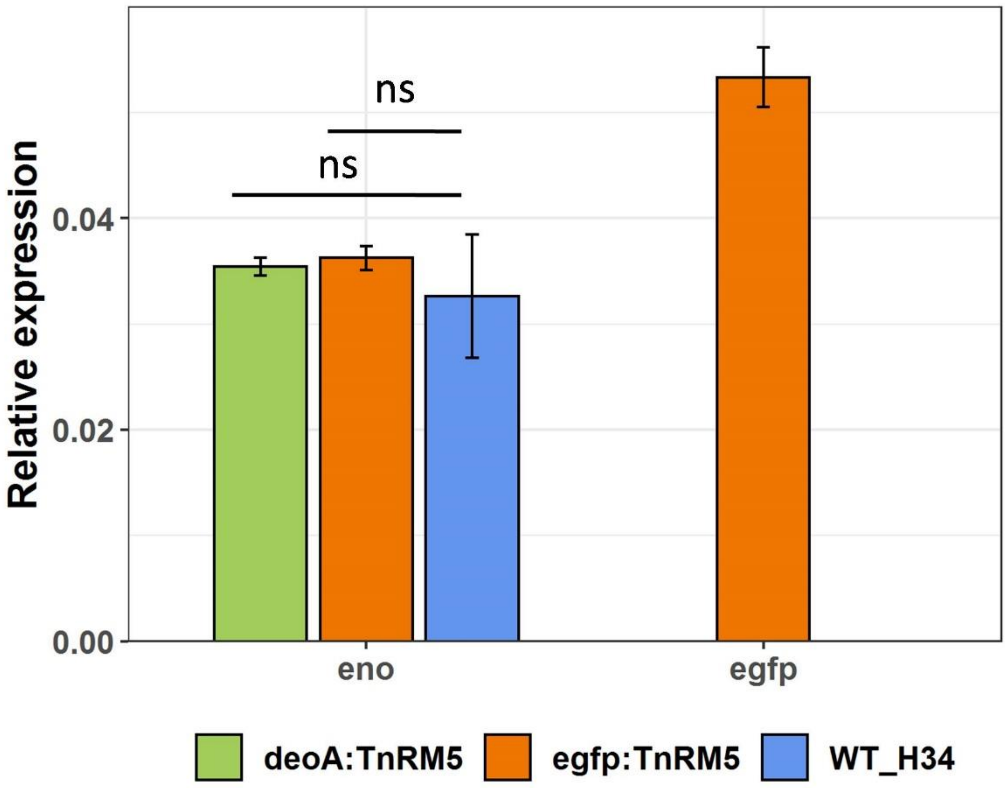

Figure S3. The relative expression level of *gfp* was determined by qRT-PCR as described in Materials. Each value represents the mean for three experiments, and error bars indicate standard error (*, significantly different from that of the wild type, $p<0.05$; ns, not significant).

Table S4, A. List of proteins upregulated in DeoA:TnRM5 *M. hominis* strain H-34 grown on liquid medium with thymidine compared to the wild type WT H-34grown on liquid medium with arginine. Scoring and quantification were performed with PDQuest8.0 software.

protein	gene	score	MCs/TCs
**Nucleosidemetabolism**			
Deoxyribosephosphatealdolase	**deoC**	161	4,09 ± 0,53
thymidinephosphorylase	**deoA**	149	2,75± 0,30
guanylatekinase	**gmk**	129	1,85± 0,24
**Sugarphosphorylation**			
Cof-type HAD-IIB family hydrolase		101	1,95± 0,25
**Celldivision**			
celldivisionprotein	**ftsZ**	102	2,05± 0,26
**Transcription**			
Transcriptiontermination/antiterminationprotein	**nusG**	42	2,15± 0,28
**Adaptation to low nutrient condition**			
DUF885 domain-containingprotein		105	2,34± 0,30
**Protease**			
prolylaminopeptidase	**pip**	152	1,85± 0,24
**Translation**			
peptidechainreleasefactor 1	**prfA**	160	2,83± 0,37
**translocasecomplex**			
Preproteintranslocasesubunit	**SecA**	244	2,33± 0,30

Table S4, B. List ofproteins downregulatedin DeoA:TnRM5 *M. hominis* strain H-34 grown on liquid medium with thymidine compared to the wild type WT H-34grown on liquid mediumwith arginine. Scoring and quantification were performed with PDQuest8.0 software.

protein	gene	score	MCs/TCs
**nucleosidemetabolism**			
Phosphopentomutase	**deoB**	44	0,45± 0,06
**argininemetabolism**			
carbamatekinase	**arcC**	53	0,44± 0,06
argininedeiminase	**arcA**	49	0,33± 0,04
**glycolisis**			
phosphopyruvatehydratase	**eno**	68	0,51± 0,07
lactatedehydrogenase	**ldh**	159	0,15± 0,02
phosphoglyceratekinase	**pgk**	158	0,42± 0,05
Glyceraldehyde-3-phosphate dehydrogenase	**gap**	79	0,11± 0,01
**celldivision**			
segregation/condensationprotein B	**scpB**	138	0,38± 0,05
GTPase	**obgE**	135	0,55± 0,07
**transcription**			
transcriptionelongationfactor	**greA**	110	0,24± 0,03
**protease**			
aminopeptidase C	**pepC**	257	0,31± 0,04
endopeptidaseLa	**lon**	249	0,28± 0,04
aminopeptidase P familyprotein		117	0,42± 0,05
M42 familypeptidase		269	0,56± 0,07
Leucylaminopeptidasefamilyprotein		220	0,37± 0,05

**translation**			
30S ribosome-bindingfactor	**RbfA**	48	0,55± 0,07
elongationfactor P	**efp**	74	0,38± 0,05
**chaperones**			
triggerfactor	**tig**	244	0,11± 0,01
molecularchaperoneDnaK	**dnaK**	224	0,14± 0,02
**phosphate-containing compound metabolic process**			
Inorganicdiphosphatase	**ppa**	48	0,44± 0,06
**Unknownfunction**			
HIT familyprotein		85	0,58± 0,08
**celldivision, chromosomeseparation**			
segregation/condensationprotein B	**scpB**	138	0,50± 0,07
**nuclease**			
TatDfamilydeoxyribonuclease	**TatD**	101	0,48± 0,06
